# Overexpression of the *Liriodendron tulipifera TPS32* gene in tobacco enhances terpenoid compounds synthesis

**DOI:** 10.3389/fpls.2024.1445103

**Published:** 2024-09-17

**Authors:** Junpeng Wu, Manli Bu, Yaxian Zong, Zhonghua Tu, Yanli Cheng, Huogen Li

**Affiliations:** ^1^ Key Laboratory of Forest Genetics & Biotechnology of Ministry of Education, Co-Innovation Center for Sustainable Forestry in Southern China, College of Forestry, Nanjing Forestry University, Nanjing, China; ^2^ College of architecture, Anhui Science and Technology University, Bengbu, Anhui, China

**Keywords:** *Liriodendron*, TPS family, genome identification, gene clone, terpenoid biosynthesis

## Abstract

*Liriodendron*, a relic genus from the Magnoliaceae family, comprises two species, *L. tulipifera* and *L. chinense*. *L. tulipifera* is distinguished by its extensive natural distribution in Eastern North America. Conversely, *L. chinense* is nearing endangerment due to its low regeneration rate. A pivotal aspect in the difference of these species involves terpenoids, which play crucial roles in plant growth and attracting pollinators. However, the complex molecular mechanisms underlying terpenoid roles in *Liriodendron* are not well understood. Terpene Synthases (TPS) genes are widely reported to play a role in terpenoid biosynthesis, hence, this study centers on *TPS* genes in *Liriodendron* spp. Employing multiple bioinformatics methods, a differential expression gene in *L. tulipifera*, *LtuTPS32*, was discerned for further functional analysis. Subcellular localization results reveal the involvement of *LtuTPS32* in chloroplast-associated processes, hence participate in terpenoid biosynthesis within chloroplasts. Heterologous transformation of the *LtuTPS32* gene into tobacco significantly elevates the levels of common terpenoid compounds, including chlorophyll, gibberellin, and carotenoids. Collectively, these findings not only underscore the role of the *LtuTPS32* gene in the biosynthesis of terpenoids but also lay a foundation for future research on interspecific differences in *Liriodendron*.

## 1 Introduction

Terpenoids constitute a remarkably vast and diverse array of plant compounds, that conform to the isoprene rule, as described by Wallach and Rutzicka (Tholl, 2015). This class comprises over 40,000 identified variations and derivatives, all sharing a fundamental C5 skeleton derived from isopentenyl diphosphate (IPP) and dimethylallyl diphosphate (DMAPP) (Hyodo et al., 2003). Terpenoids are categorized based on the number of C5 units, ranging from hemiterpenes (C5) to monoterpenes (C10), sesquiterpenes (C15), diterpenes (C20), triterpenes (C30), and others (Campos et al., 2001).

**Figure 1:**
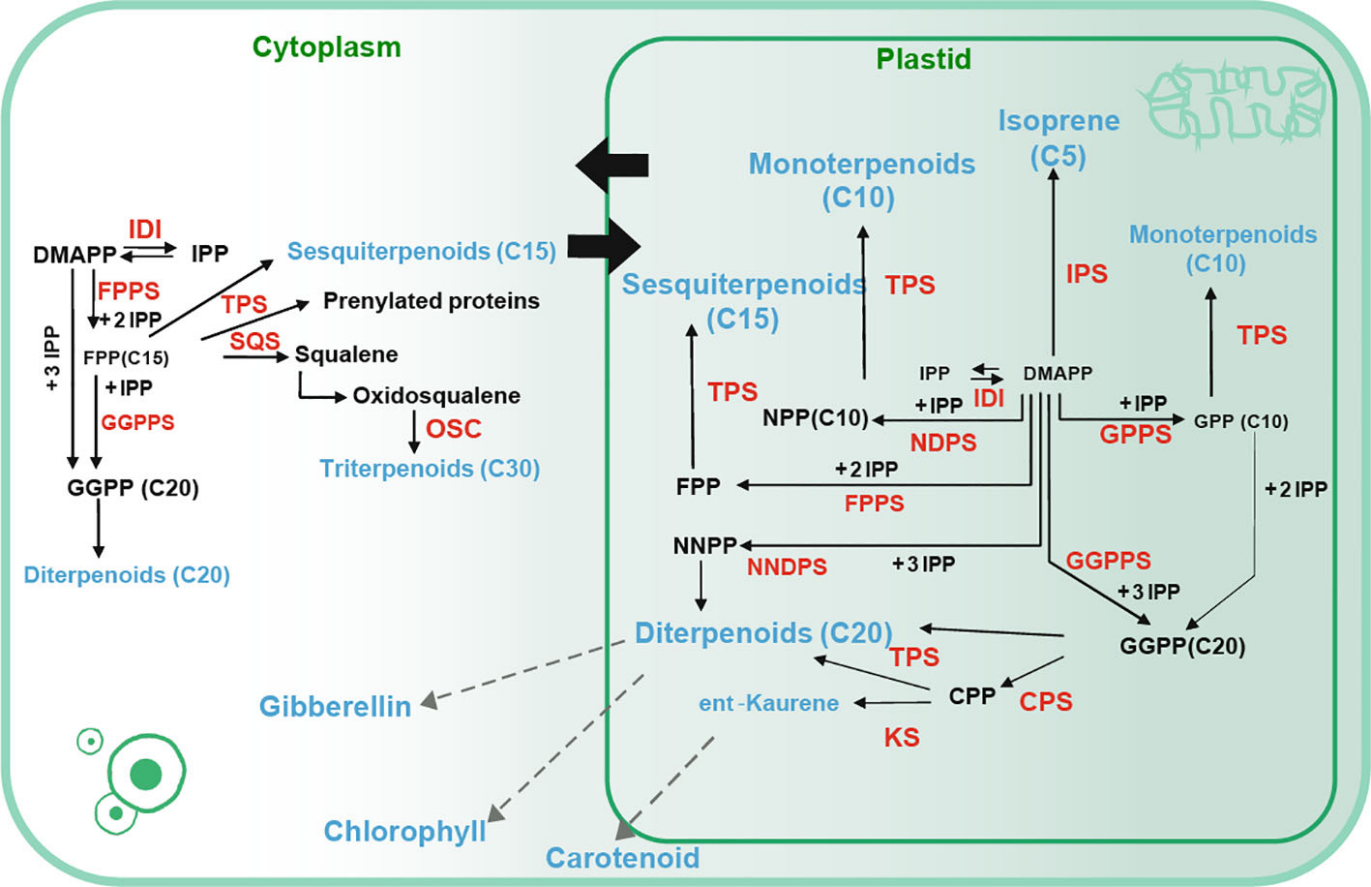
Terpene biosynthesis pathway (Tholl, 2015). Solid arrows represent a single step whereas dashed arrows represent multiple biosynthetic reactions. Catalytic products are denoted in blue font, and enzymes involved in catalysis are indicated in red font. IDI: isopentenyl diphosphate isomerase, DMAPP: dimethylallyl pyrophosphate. IPP: isopentenyl pyrophosphate, DMAPP: dimethylallyl pyrophosphate, FPPS: Farnesyl diphosphate synthase, TPS: terpene synthase, OSC: (3S)-2,3-oxidosqualene cyclase, SQS: squalene synthase, GPPS: Geranyl pyrophosphate synthase, CPP: *ent*-copalyl diphosphate, CPS: copalyl diphosphate synthase, KS: *ent*-kaurene synthase, NPP: Neryldiphosphate, NNPP: Neryldiphosphate synthase.

These compounds are predominantly synthesized through two distinct biosynthetic pathways: the cytosolic mevalonate (MVA) pathway, which primarily generates precursors for sesquiterpenes, polyprenols, plant sterols, and brassinosteroids, and the plastidial methylerythritol phosphate (MEP) pathway, which primarily furnishes precursors for hemiterpenes, monoterpenes, and diterpenes. Notably, these pathways are interconnected, facilitating substrate exchange within the plant’s metabolic network (**Figure 1**) (Lichtenthaler, 1999; Schuhr et al., 2003; Sapir-Mir et al., 2008). The complex synthesis of terpenoids involves various enzymes, culminating in the production of key precursors such as geranyl diphosphate (GPP), farnesyl diphosphate (FPP), and geranylgeranyl diphosphate (GGPP), which serve as the building blocks for monoterpenes, sesquiterpenes, and diterpenes, respectively (Takahashi and Koyama, 2006).

Beyond their structural roles, terpenoids are integral to plant growth, serving as photosynthetic pigments (carotenoids), electron carriers (side-chains of ubiquinone and plastoquinone), and regulators of growth and development (gibberellins, abscisic acid, strigolactones, brassinosteroids, cytokinins). They are also crucial for protein glycosylation and membrane structure (Estévez et al., 2001; Rodríguez-Concepción, 2004; Enfissi et al., 2005; Hong et al., 2012). Besides, terpenoids assume a crucial function in plant-environment and plant-plant interactions, offering essential defenses against various stressors. For instance, linalool, a primary volatile compound in *Psittacanthus calyculatus* flowers, possesses innate attractiveness to a wide array of insects, significantly promoting the reproductive throughout its flowering stages (Xiao, 2019). Similarly, *Quercus variabilis* releases monoterpenes like myrcene and limonene under high temperatures, enhancing photosynthesis and photorespiration rates, thereby mitigating heat stress (Peñuelas and Llusià, 2002).

Given the profound impact on plants, the study of terpenoid compounds has garnered extensive attention. The diversity and complexity of plant terpenoids are largely attributed to the action of terpene synthases (TPS), enzymes encoded by a substantial gene family. These genes are categorized into 8 subfamilies, TPSa-h. The universality of their products, terpenoids, have been found across plant species (Chen et al., 2011). However, the specific activities of TPS enzymes are challenging to delineate due to their propensity for the random rearrangement of carbon cation intermediates. These enzymes also exhibit remarkable flexibility in acquiring new catalytic properties through minor structural alterations. For instance, *AtTPS-Cin* in *Arabidopsis thaliana* can produce a diverse array of sesquiterpenes. Besides, terpenoids may undergo further diversification through secondary modifications like hydroxylation, peroxidation, methylation, acylation, glycosylation, or cleavage, thereby increasing their structural complexity and functional diversity (Tholl et al., 2005; Degenhardt et al., 2009; Christianson, 2017). Recent studies have successfully identified and catalogued the *TPS* gene family in various plant species, revealing significant differences in the number of *TPS* genes among species like *A. thaliana*, *Magnolia biondii*, *Cinnamomum kanehirae*, and *Vitis vinifera* (Aubourg et al., 2002; Martin et al., 2010; Chaw et al., 2019; Dong et al., 2021).

*Liriodendron*, belonging to the Magnoliaceae family, is a genus of particular interest, comprising two species: *L. tulipifera* and *L. chinense*. While *L. tulipifera* is known for its multiple uses such as timber production and urban landscaping, *L. chinense* is at risk of endangerment due to its low regeneration rate (He and Hao, 1999; Li, 2013). Previous studies have shown significant interspecific variation in terpenoid compounds between these two species, and identified TPS family in an old version of the *L. chinense* genome (Zhang et al., 2022; Cao et al., 2023). However, the molecular mechanism underlying these differences in terpenoid synthesis in *Liriodendron* is not well understood. Hence, given the critical roles of *TPS* genes in plant growth, our study uses the genome of *L. tulipifera* and latest annotation of *L. chinense* genome to identify TPS family and focuses on the interspecific variation in terpenoid biosynthesis in *Liriodendron* (Wu et al., 2023). Overall, the results of our study serve as a valuable reference for further exploration of *TPS* gene function and will aid future studies of terpenoids synthesis in *Liriodendron*.

## 2 Materials and Methods

### 2.1 Plant materials and growing conditions

*Liriodendron* samples were collected from Xiashu Forest Station at Jurong county, Jiangsu province, China (S32°37′, E118°57′). The seed sources for *L. chinense* and *L. tulipifera* were obtained from Wuyi Mountain, Fujian Province, China, and the State of Georgia, USA, respectively. Various floral tissues, including petals, stamens, pistils, and flowers were harvested and immediately preserved in liquid nitrogen before storage at -80°C for further analysis.

Tobacco (*Nicotiana tabacum*) was used for transgenic experiments. Seeds were sterilized through a sequential treatment beginning with 75% (v/v) alcohol for 30 seconds, followed by a 15-minute immersion in sodium hypochlorite (NaClO), and rinsed five times with double-distilled water. The sterilized seeds were sown on half-strength Murashige and Skoog (MS) medium supplemented with 30 g/L sugar and incubated at 4°C in darkness for two days (Murashige and Skoog, 1962). Following this, the seeds were transferred to a growth chamber with a 16-hour light/8-hour dark cycle at 23°C for germination. Seedlings were transferred to MS medium for an additional 20 days of growth before genetic transformation experiments.

### 2.2 Identification and characterization of TPS family members in *Liriodendron*

Genome data for *Liriodendron* were retrieved from the NCBI (https://www.ncbi.nlm.nih.gov/) and JGI (https://genome.jgi.doe.gov/portal/) databases and the latest annotation of *L. chinense* genome was used as reference. Hidden Markov Model (HMM) profiles of TPS family domains (PF01397 and PF03936) were obtained from the Pfam database. We employed HMMER software (version 3.0) to scan the *Liriodendron* protein sequences for these domains, setting the significance threshold at 1e-5 (Potter et al., 2018; El-Gebali et al., 2019). Protein sequences were also searched using Blast software, with published *Cinnamomum kanehirae* TPS protein sequences as a reference (Chaw et al., 2019). Alignments against the *Liriodendron* protein sequences were conducted with the same threshold of significance. The obtained protein sequences were further compared to SMART databases (https://smart.embl.de/) for validation (Letunić et al., 2021). Incomplete and redundant sequences were manually excluded.

### 2.3 Chromosomal localization and physicochemical properties analysis of the TPS family in *Liriodendron*

Chromosomal distribution of *TPS* genes in *Liriodendron* was visualized using TBtools software based on GFF file information. and renamed them as *TPS1*-*TPS70*. To further characterize these genes, the ExPASy online tool (https://web.expasy.org/protparam/) was employed for predicting the physicochemical properties of the TPS proteins. These analyses included the assessment of molecular weight (MW), theoretical isoelectric point (pI), and hydrophilicity (Wilkins et al., 1999; Chen C. et al., 2020).

### 2.4 Phylogenetic analysis of TPS members

Phylogenetic analysis was conducted using sequences from *Abies grandis*, *Arabidopsis thalian*a, *Selaginella moellendorffii*, and *Cinnamomum kanehirae*. These sequences were aligned employing MAFFT software (version 7.310) using its default settings. Then, constructed the phylogenetic tree with Fasttree software (version 2.1.0), applying 1,000 bootstrap replicates with Maximum Likelihood (ML) method. The phylogenetic tree was further modified using the interactive tool iTOL (Price et al., 2010; Katoh and Standley, 2013; Letunic and Bork, 2021). To investigate the motif patterns and the exon-intron structure of TPS members, the MEME and GSDS2.0 were employed, respectively (Bailey et al., 2015; Hu et al., 2015). Protein similarity assessments were conducted using Blastp software. Additionally, we analyzed the *cis*-regulatory elements in the TPS promoter regions by extracting the 2000 bp sequence upstream of the start codon (ATG) and analyzing it through the PlantCARE database (https://bioinformatics.psb.ugent.be/webtools/plantcare/html/) (Lescot et al., 2002).

### 2.5 Expression patterns of *TPS* genes

We investigated the expression levels of *TPS* family genes in *Liriodendron* by analyzing publicly available transcriptomic data (NCBI, PRJNA559687). The FPKM values of *TPS* genes were extracted to facilitate further functional analysis.

### 2.6 RNA extraction and spatiotemporal expression profiling via RT-qPCR for *TPS* genes

RNA was extracted using the SteadyPure Plant RNA Extraction Kit (AG21019, Accurate Biotechnology, Hunan, Co., Ltd.), and its quality was verified using a NanoDrop 2000 spectrophotometer. For cDNA synthesis, 500 ng of RNA was used.

To profile the expression of *TPS* genes, RT-qPCR was conducted, using *Actin97* as the internal control (Tu et al., 2019). The RT-qPCR thermal cycling conditions followed those recommended in the SYBR Green Premix Pro Taq HS qPCR Kit (AG11701, Accurate Biotechnology, Hunan, Co., Ltd.). Expression levels were quantified using the 2^−ΔΔCT^ method (Livak and Schmittgen, 2001). To ensure data reliability, each experiment included three biological and technical replicates. Primer sequences are listed in **Supplementary Table S1**.

### 2.7 Full-length cDNA cloning of *LtuTPS32* and heterologous transformation

The coding sequence of *LtuTPS32* was isolated from the *L. tulipifera* genome, yielding a 1728-bp open reading frame (ORF). This gene was cloned into a modified pBI-121 vector, pre-treated with XbaI and BamHI QuickCut enzymes (Takara Biomedical Technology, Dalian, China). The recombinant vector was introduced into the *Agrobacterium tumefaciens* GV105 strain for tobacco transformation via the leaf-disc method. In this process, wild-type tobacco leaves, cultivated for about 30 days, were trimmed around the edges and subsequently incubated on solid MS medium at a consistent temperature of 25°C, in darkness, for two days. The *A. tumefaciens*, carrying the modified vector, was then activated in liquid MS medium for half an hour to prepare the infection solution. Tobacco leaves were submerged in this solution for ten minutes. After infection, the leaves were placed in darkness at 25°C for an additional two days, followed by exposure to a cycle of 16 hours of light and 8 hours of darkness at the same temperature. Calli emerging at the leaf edges were isolated and cultured on MS medium supplemented with kanamycin (100 mg/L). After 20 days, PCR was used to screen positive tobacco, which were then grown to maturity.

### 2.8 Promoter cloning and subcellular localization of LtuTPS32

We extracted the promoter sequence (2000 bp upstream of the start codon) of *LtuTPS32*, and submitted it to PlantCARE website (https://bioinformatics.psb.ugent.be/webtools/plantcare/html/). And we fused it to the pBI121-GUS-tagged vector, introduced the vector into *Agrobacterium tumefaciens* GV3101 strain, and transiently transformed the *A. tumefaciens* into tobacco leaves. Leaves transformed with the empty pBI121-GUS vector were used as a positive control, while wild-type leaves served as negative controls. Tobacco leaves were treated with 10 mmol/L MeJA,100 μmol/L ABA solution and H2O, respectively. We treated different leaves on three tobacco plants and took five leaf discs from each leaf. After incubating them with 16 h light and 8 h darkness for 2 days, the treated leaves were subjected to GUS staining. Then, decolorization was performed using 70% ethanol until the samples lost green color.

We cloned the full-length coding sequences of *LtuTPS32* into pCAMBIA1305 vector containing the GFP tag, and the transgene construct was introduced into *A. tumefaciens* GV3101 strain, then transiently transformed the constructs into tobacco leaves, and observed the results using confocal.

### 2.9 Determination of chlorophyll, carotenoids, and gibberellins

We used the spectrophotometry to measure the contents of chlorophyll and carotenoids, and the method referred to previous described (Yang et al., 2021), and the content of gibberellins was measured using an ELISA kit (Shanghai Huding Biological Technology Co., LTD).

## 3 Results

### 3.1 Identification, characterization and chromosomal localization of TPS family members in *Liriodendron*

Utilizing HMMER software, we scan the genome of *L. chinense* and *L. tulipifera.* In *L. chinense*, 67 TPS members were identified. A subsequent Blast method was employed to scan the genome, and yielded 87 TPS sequences. After aligning these sequences that were gotten form BLAST method against SMART and NCBI databases, we excluded proteins lacking TPS domains and those shorter than 200 amino acids, ultimately identifying 70 TPS members in the *L. chinense* genome. Employing a similar method, we also identified 70 TPS members in the *L. tulipifera* genome. Using the latest genome annotations and BLAST results, we discovered an additional 12 *TPS* genes in *L. chinense* compared with previous reports (Cao et al., 2023; Wu et al., 2023).

To elucidate the chromosomal distribution and potential evolutionary relationships of the TPS family in *Liriodendron*, we conducted a chromosomal localization assay (**Supplementary Figures S1**, **S2**). In *L. chinense*, 49 *TPS* genes are distributed across chromosomes 1, 3, 6, 12, 14, and 19, with an additional 21 *TPS* genes not assigned to any specific chromosome. Chromosome 1 harbors the largest number of *TPS* genes (23), whereas chromosome 6 contains just one. In *L. tulipifera*, 65 *TPS* genes are distributed across chromosomes 01, 03, 06, 10, 14, 15, and 19, with five genes unassigned to any chromosome. Chromosome 10 has the densest accumulation, hosting 34 *TPS* genes, whereas chromosomes 14 and 15 each has only one. For ease of further analysis, we renamed these genes as *LcTPS1* to *LcTPS70* and *LtuTPS1* to *LtuTPS70*, respectively (**Supplementary Tables S2**, **S3**).

Additionally, we analyzed the physicochemical properties of TPS proteins using the ExPASy platform, with results detailed in **Supplementary Tables S2** and **S3**. In *L. chinense*, the largest TPS protein, LcTPS34, measures 96.91 kDa and comprises 852 amino acids, while the smallest, LcTPS70, is 27.70 kDa and 241 amino acids. Their isoelectric points (pI) range from 4.86 (LcTPS48) to 9.18 (LcTPS50), all exhibiting hydrophilic properties. Similarly, in *L. tulipifera*, the largest protein (LtuTPS63) and the smallest (LtuTPS17) display comparable diversity in size and hydrophilicity, with pI values ranging from 4.88 (LtuTPS66) to 7.67 (LtuTPS11).

### 3.2 Phylogenetic analysis of TPS members in *Liriodendron*

**Figure 2:**
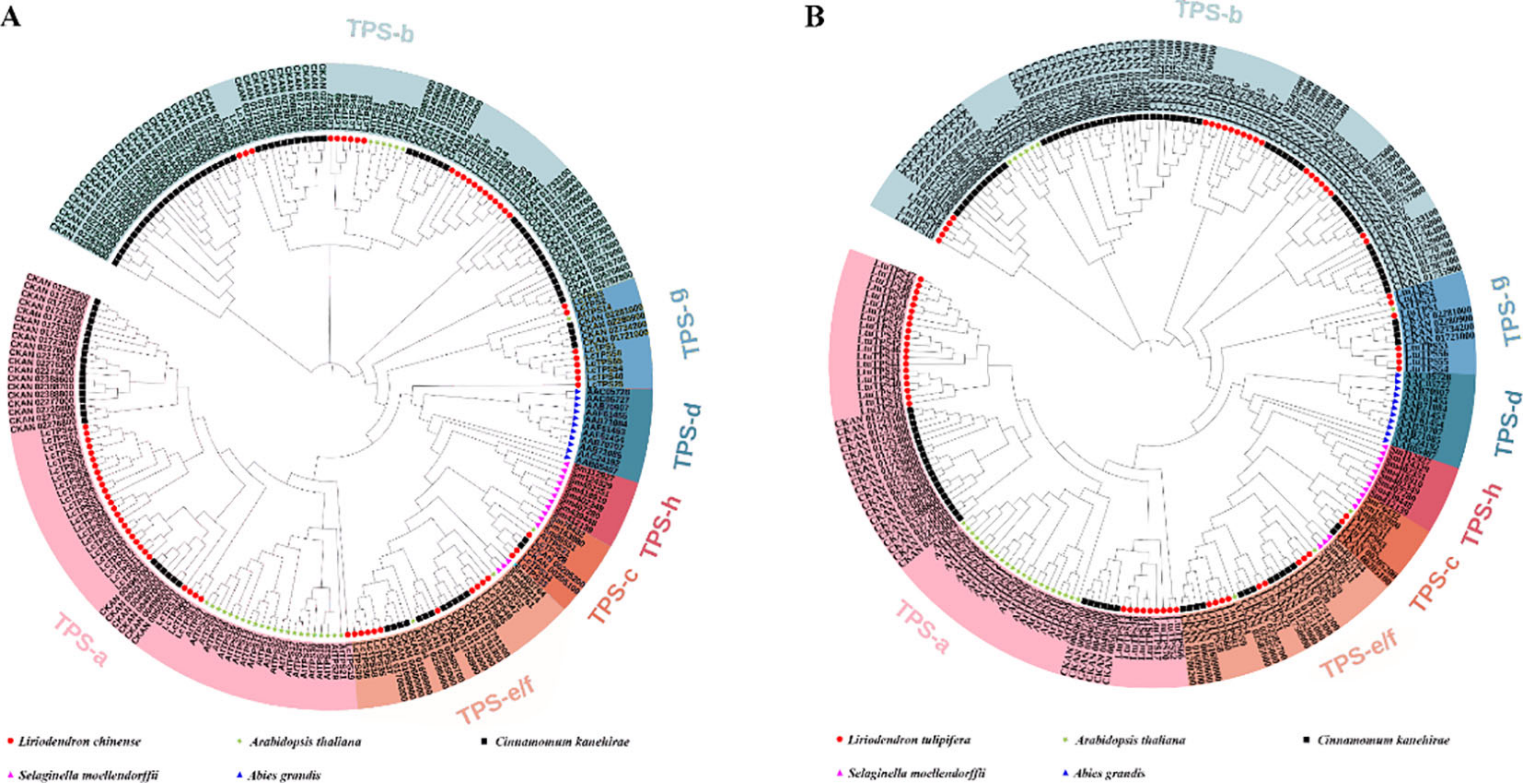
The phylogenetic tree of *Liriodendron* TPS members. **(A)** The red circle, green star, black square, pink triangle and blue triangle represent *Liriodendron chinese*, *Arabidopsis thaliana*, *Cinnamomum kanehirae*, *Selaginella moellendorffii* and *Abies grandis*, respectively. **(B)** The red circle, green star, black square, pink triangle and blue triangle represent *L. tulipifera*, *A. thaliana*, *C. kanehirae*, *S. moellendorffii* and *A. grandis*, respectively. Different colored backgrounds represent different subfamilies.

To further investigate the evolutionary relationships and classification among *TPS* genes, we aligned TPS protein sequences from *A. grandis*, *A. thaliana*, *S. moellendorffii*, and *C. kanehirae*, alongside TPS members identified in *L. tulipifera* and *L chinense* (**Figures 2A, B**). This analysis showed that TPS members were distributed across seven subfamilies: TPS-a, TPS-b, TPS-c, TPS-d, TPS-e/f, TPS-g, and TPS-h. In *L. chinense* (**Figure 2A**), *TPS* genes were categorized into five subfamilies, with the TPS-a subfamily is the largest with 28 members, followed by TPS-b with 21 members. The other subfamilies, TPS-c, TPS-e/f, and TPS-g, consist of 3, 10, and 8 members, respectively. *L. tulipifera* showed a similar distribution, with the largest being TPS-a (29 members), followed by TPS-b (23 members). The TPS-c, TPS-e/f, and TPS-g subfamilies contain 2, 9, and 7 members, respectively. Notably, the TPS-d and TPS-h subfamilies were exclusive to gymnosperms and *S. moellendorffii*, respectively.

**Figure 3:**
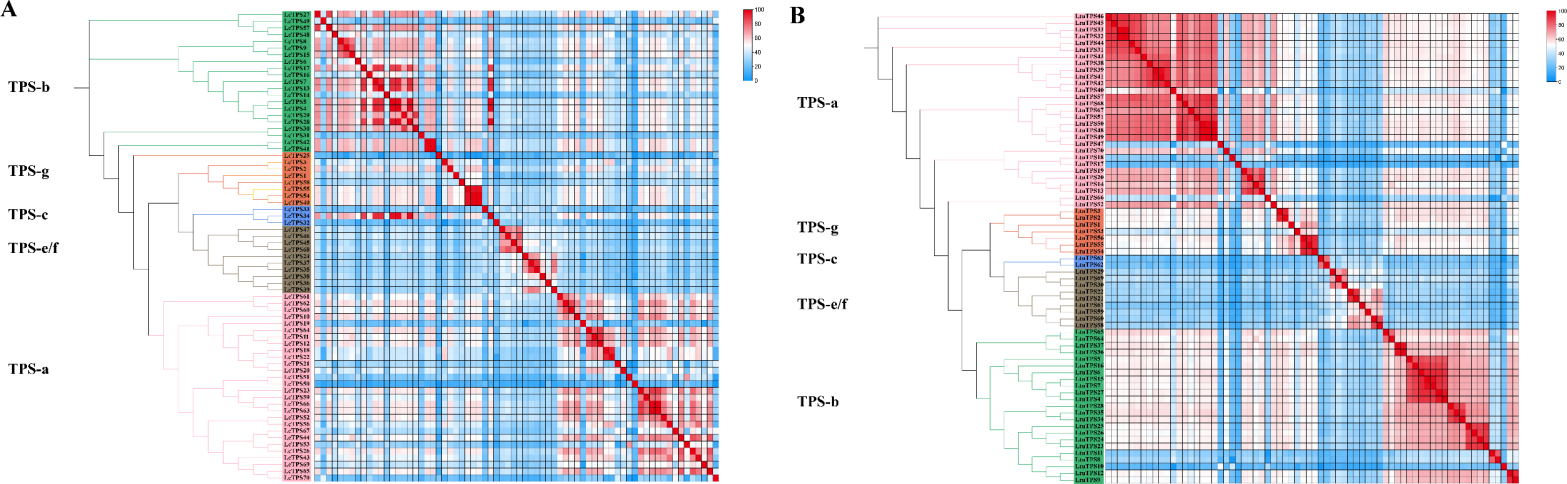
**(A)** The phylogenetic tree and heat map of protein similarity matrix of *Liriodendron chinese* TPS family. **(B)** The phylogenetic tree and heat map of protein similarity matrix of *L. tulipifera* TPS family. Different colored backgrounds represent different subfamily classifications, and the phylogenetic trees are drawn. The similarities are visualized and different colored squares represent protein similarity.

Tandem duplication, a key evolutionary mechanism promoting rapid gene family expansion and diversification, is prevalent in TPS families and plays a pivotal role in adapting metabolic pathways to ecological interactions (Hoeven et al., 2000; Pichersky and Gang, 2000; Kliebenstein et al., 2001; Köllner et al., 2004). To further explore the expansion and evolutionary dynamics within the *Liriodendron* TPS family, we computed a protein similarity matrix visualized through heatmaps (**Figures 3A, B**). Proteins sharing over 75% similarity, marked by deep red squares in the heatmaps, indicate tandem repeats. In *L. chinense*, we identified 59 pairs of tandem repeat sequences with similarities ranging from 75.27% to 99.65%, predominantly within the TPS-a and TPS-b subfamilies. Similarly, in *L. tulipifera*, 177 pairs of tandem repeat sequences were identified, with similarities between 75.09% and 99.64%, also mainly in the TPS-a and TPS-b subfamilies, suggesting a robust mechanism for adaptation and diversification within this genus.

### 3.3 *TPS* gene expression patterns in the floral organs of *Liriodendron*

**Figure 4:**
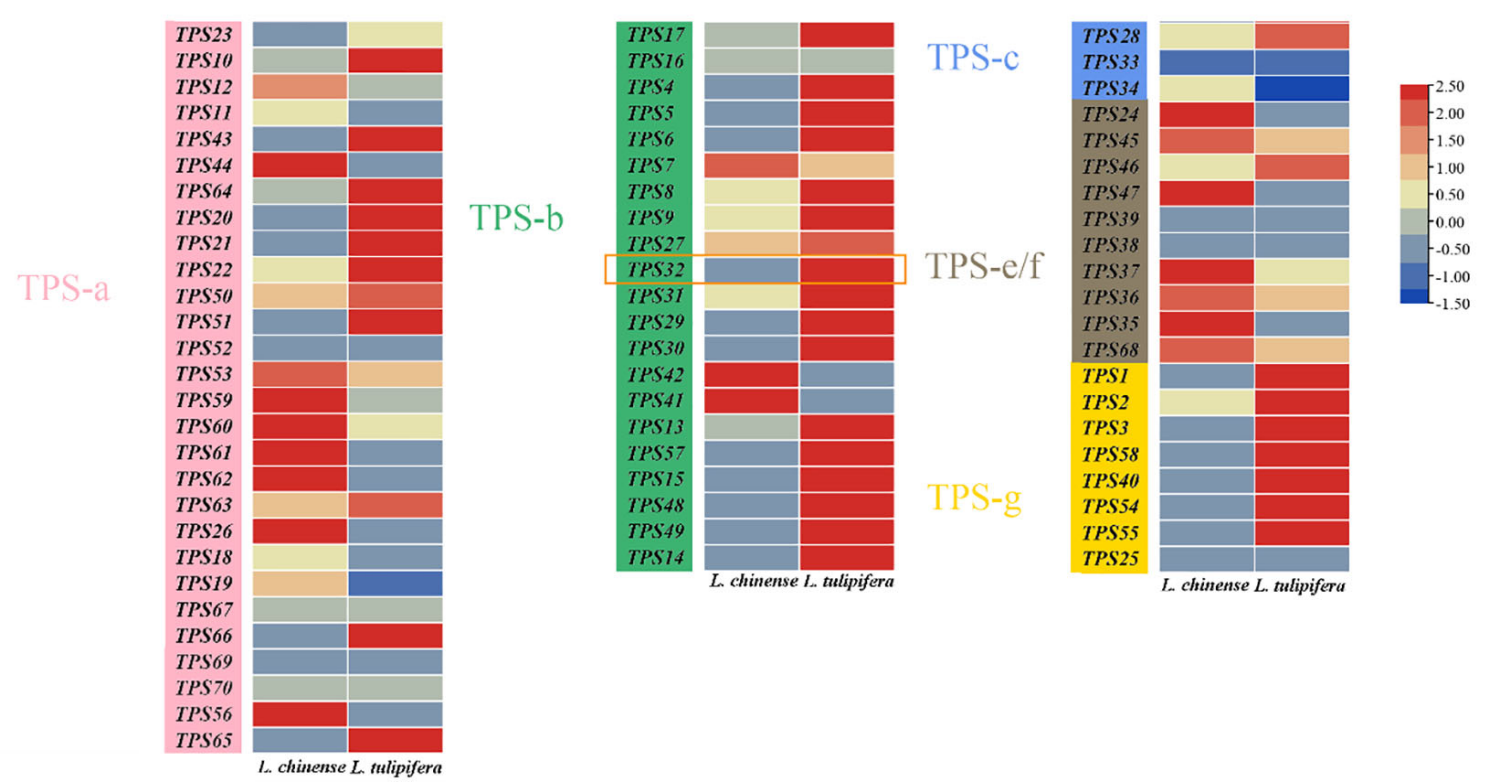
The heat map of *TPS* genes expression patterns. The expression levels of *TPS* genes across different tissues of *Liriodendron chinense* and *L*. *tulipifera* based on FPKM values from public transcriptomic data. The expression level is visualized in a heatmap format, and different colors represent different expression levels. The orange rectangular in the figure represents the *LtuTPS32* gene.

Investigating gene expression patterns is crucial for guiding gene cloning and functional analysis. Therefore, we analyzed transcriptomic data from the floral organs of *Liriodendron*, visualized in a heat map (**Figure 4**). This analysis highlighted notable disparities in *TPS* genes expression across different species. Within the TPS-a subfamily, 12 genes were more highly expressed in *L. chinense*, compared to 13 in *L. tulipifera*, with 4 genes undetected. In the TPS-b subfamily, 3 genes exhibited higher expression in *L. chinense*, whereas 17 genes were more prominent in *L. tulipifera*, with 1 gene undetected. In the TPS-c subfamily, 1 gene each showed higher expression in *L. chinense* and *L. tulipifera*, with 1 gene undetected. For the TPS-e/f subfamily, 7 genes expressed higher in *L. chinense*, one in *L. tulipifera*, and 2 genes remained undetected. In the TPS-g subfamily, 7 genes were predominantly expressed in *L. tulipifera*, with one gene undetected. These undetected *TPS* genes might be expressed in other tissues or could have lost functionality through evolutionary processes. The differential expression was primarily observed in the TPS-b, TPS-e/f, and TPS-g subfamilies. Notably, in angiosperms, the TPS-b and TPS-g subfamilies are implicated in the synthesis of monoterpenes, potentially accounting for the high monoterpene emission observed in *L. tulipifera*.

### 3.4 Cloning and expression profiles of *LtuTPS32* in *Liriodendron*

Our transcriptome analysis showed that the expression of TPS-b subfamily was highly biased, and previous study displayed significant interspecies differences in monoterpene compounds (Zhu, 2020). However, there is a scarcity of reports on terpenoids biosynthesis in *Liriodendron*, and the potential mechanism underpinning these terpenoids remains unclear. Research on *Arabidopsis thaliana* has demonstrated that the *TPS02* gene significantly influences the levels of terpenoid volatiles across ecotypes (Huang et al., 2010). We hypothesize that this gene might have a similar function in *Liriodendron* species. Therefore, in order to further investigate the potential mechanisms, we chose its homologous gene, *TPS32*, as the focus of our study, and successfully cloned the *TPS32* gene by utilizing *L. tulipifera* cDNA as a template.

**Figure 5:**
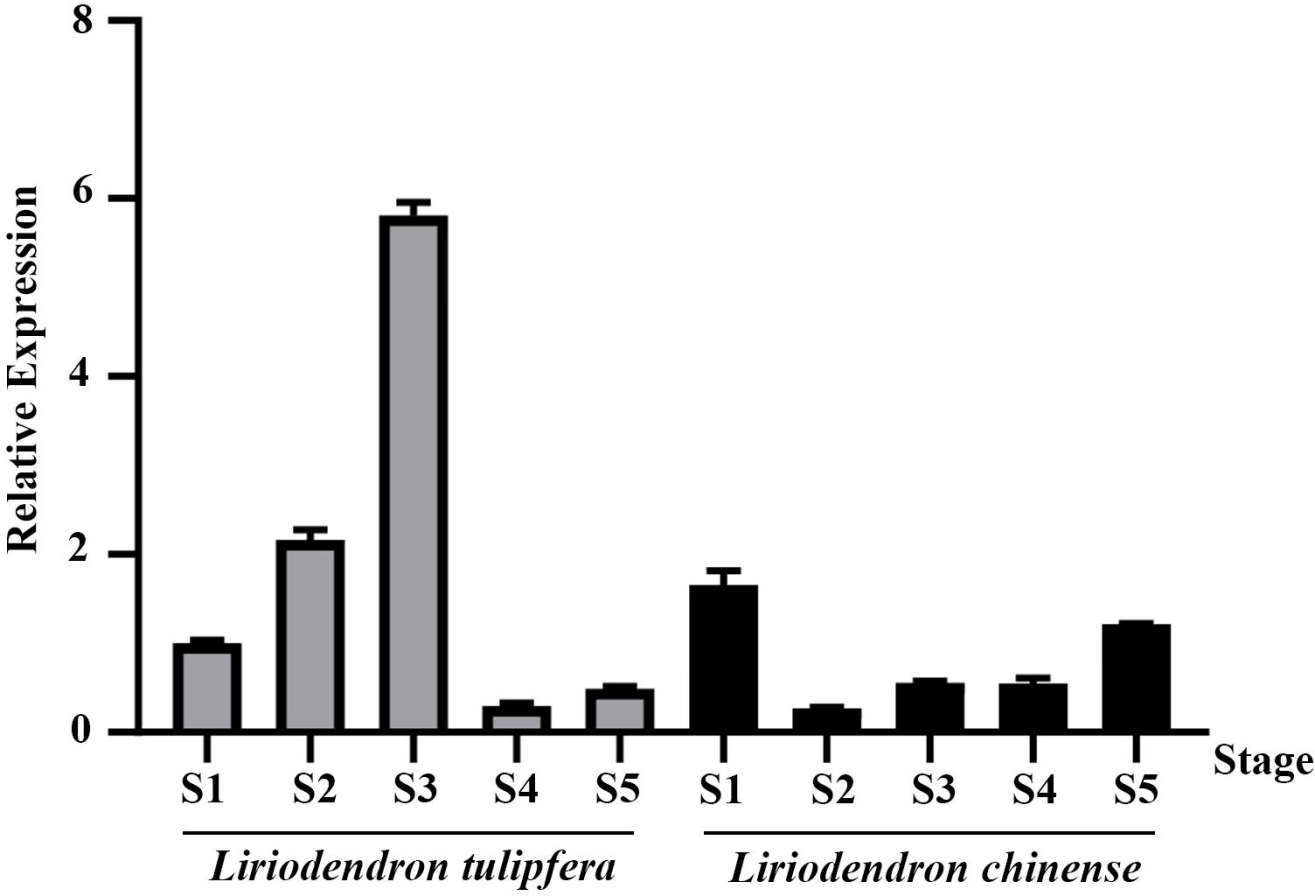
The relative expression of *TPS32* gene in five flowering stages of *Liriodendron tulipifera* and *Liriodendron chinense*, S1, S2, S3, S4 and S5 respectively represent flower bud stage, budding stage, germination stage, blooming stage and senescence stage. Error bars on the graph indicate the standard deviation for each triplicate treatment.

We analyzed previous published *Liriodendron* transcriptome data (Tu et al., 2021) and found that this gene was highly expressed in flowers. To precisely delineate the expression pattern of *TPS32*, we performed RT-qPCR analysis across various tissues (pistils, stamens, and petals) and developmental stages (S1 to S5) in *Liriodendron*. As depicted in **Figure 5**, expression of *TPS32* peaked during the S3 stage in *L. tulipifera*, showing a 5.8-fold increase over the S1 stage. Conversely, in *L. chinense*, the highest expression was noted at the S1 stage, at levels 1.65 times higher than *L. tulipifera* during the same stage.

**Figure 6:**
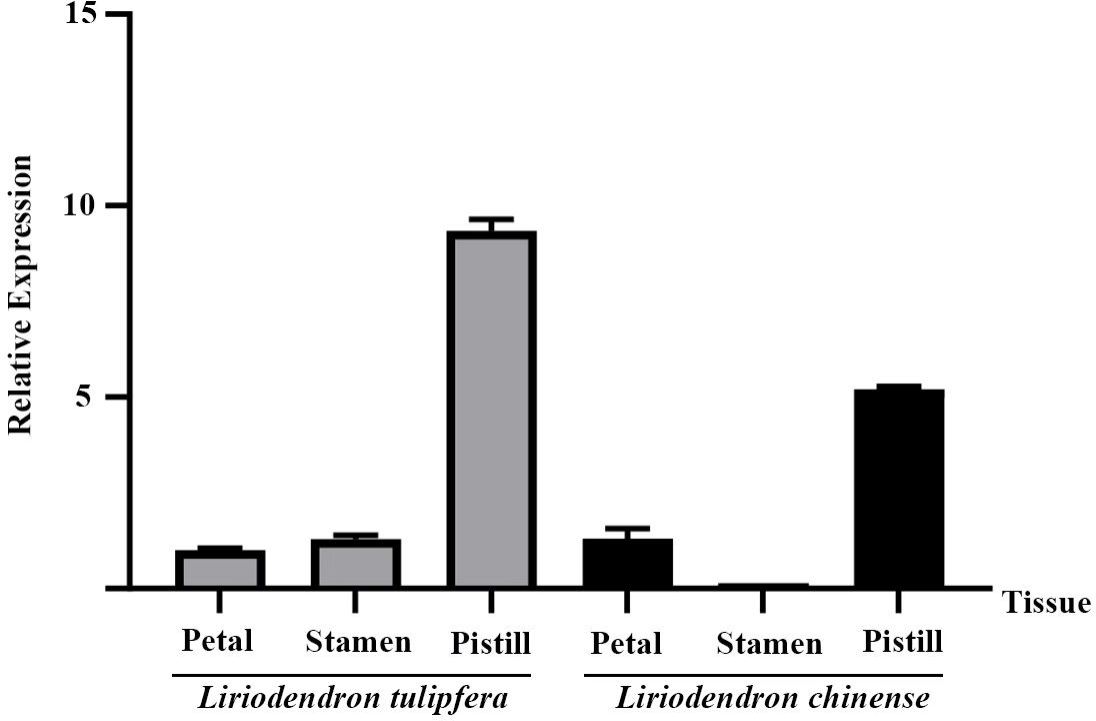
The relative expression of *TPS32* gene in petal, stamen and pistil of *Liriodendron tulipifera* and *L. chinense*. Error bars on the graph indicate the standard deviation for each triplicate treatment.

Further analysis across different floral tissues (**Figure 6**) revealed that in both species, *TPS32* was most abundantly expressed in the pistils, reaching levels that were 9.3 times and 5.2 times higher than those observed in the petals of *L. tulipifera*, respectively. The lowest expression levels were found in the petals of *L. tulipifera* and in the stamens of *L. chinense*, highlighting tissue-specific expression patterns that may contribute to distinct ecological and reproductive strategies.

### 3.5 Promoter cloning and subcellular localization of LtuTPS32

**Figure 7:**
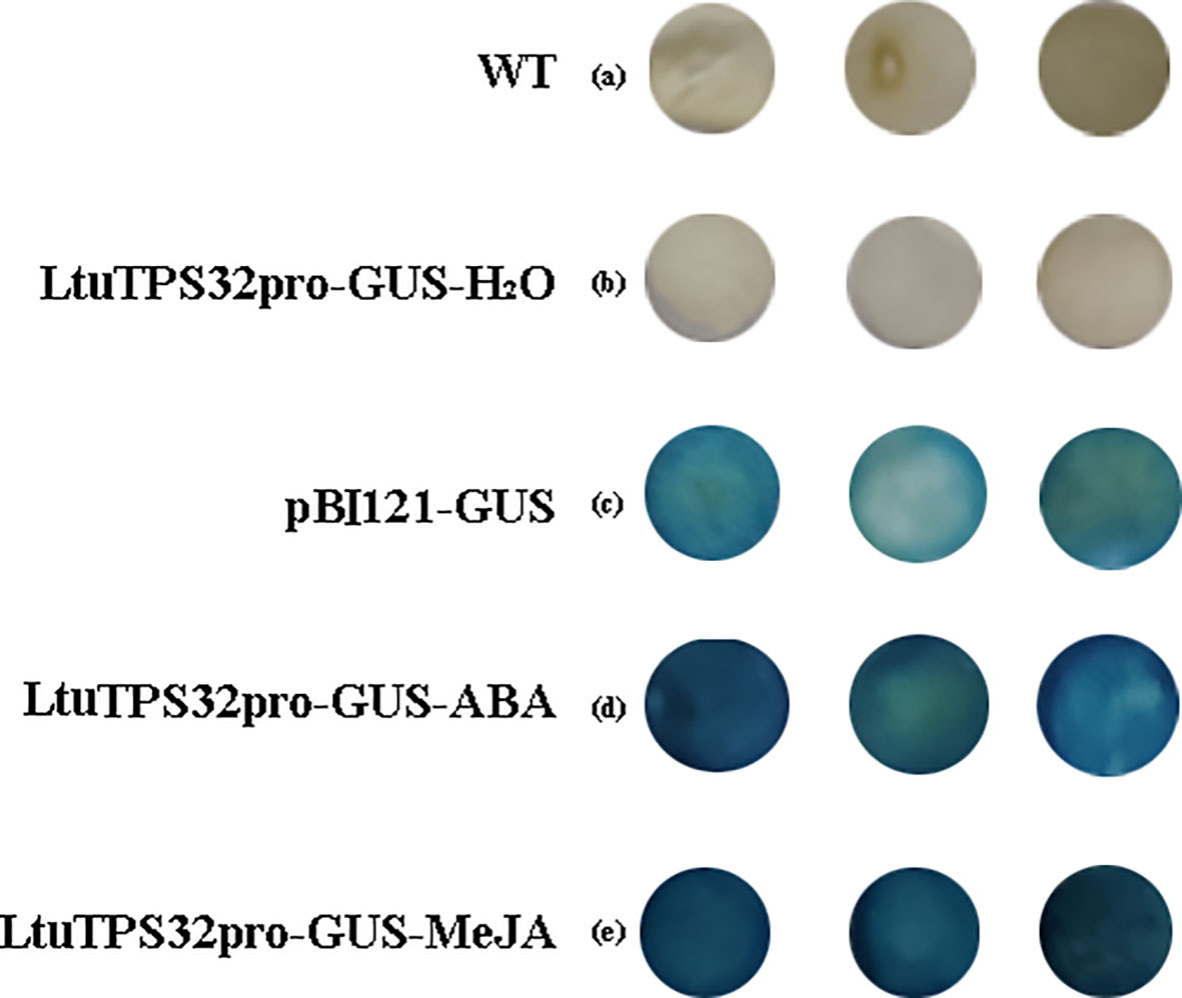
The GUS staining of LtuTPS32 promoter. **(A)** Untreated tobacco leaves. **(B)** Tobacco leaves treated with H_2_O. **(C)** Tobacco leaves that transiently transformed the pBI121-GUS plasmid. **(D)** Tobacco leaves treated with ABA. **(E)** Tobacco leaves treated with MeJA.

The promoter region plays a crucial role in regulating gene transcription and mediating environmental responses. To explore the regulatory dynamics of *LtuTPS32*, we cloned its promoter and identified multiple cis-regulatory elements: three abscisic acid-responsive elements (ABREs) and ten MYC elements, which are known to respond to abscisic acid (ABA) and methyl jasmonate (MeJA), respectively. We fused its promoter to a *β*-glucuronidase (GUS) reporter gene (*LtuTPS32p::GUS*). Using an *Agrobacterium*-based transient expression system, we introduced this fused vector into tobacco leaves. Subsequent treatments with H2O, ABA, and MeJA followed by GUS staining revealed that discs treated with ABA and MeJA exhibited a significantly deeper blue color compared to the control (pBI121-GUS) (**Figure 7**). This enhanced staining indicates a strong inducible activity of the LtuTPS32 promoter by ABA and MeJA, suggesting that environmental cues via these phytohormones could activate *LtuTPS32* expression.

**Figure 8:**
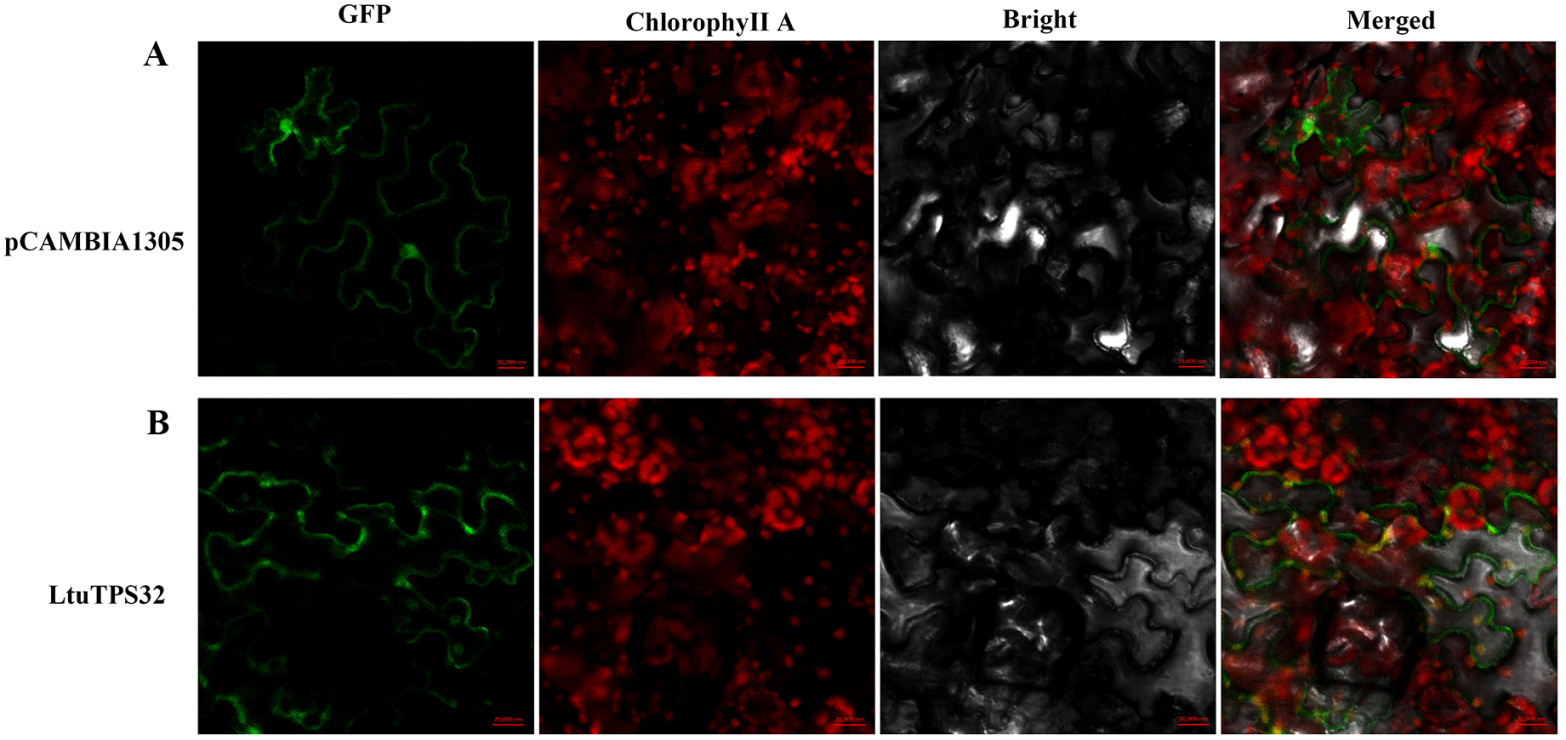
The subcellular localization of LtuTPS32, **(A)** The fluorescence and excitation of pCAMBIA1305 protein under different excitation conditions. **(B)** The fluorescence and excitation of LtuTPS32 protein. Localization analysis using empty pCAMBIA1305 vector and *35S:: LtuTPS32*-GFP, respectively. Scale bar, 20,000 nm.

Furthermore, to elucidate the functional implications of *LtuTPS32*’s expression patterns, we conducted a subcellular localization study. The LtuTPS32 protein, tagged with a green fluorescent protein (GFP), was transiently expressed in *L. tulipifera* protoplasts. Observations under a confocal microscope showed pronounced localization of the LtuTPS32-GFP fusion within the chloroplasts (**Figure 8**). This specific localization underscores the potential role of LtuTPS32 in chloroplast-associated processes, likely contributing to terpenoid biosynthesis within this organelle, pivotal for the plant’s metabolic and stress response mechanisms.

### 3.6 Overexpression of *LtuTPS32* enhanced terpenoid biosynthesis in transgenic tobacco

**Figure 9:**
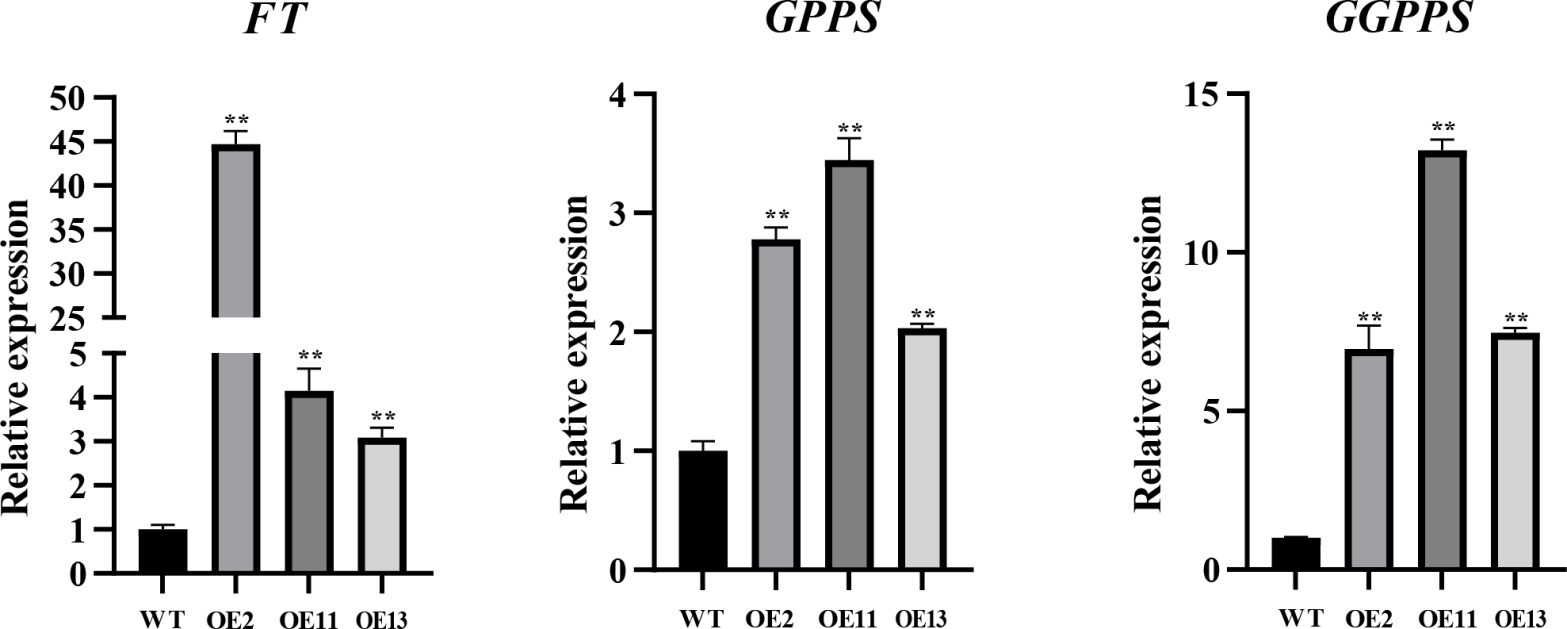
RT-qPCR analysis of transgenic and wide-type plants. The relative expression level of *FT*, *GPPS*, and *GGPPS* genes are calculated using the 2^−ΔΔCT^ method. Error bars on the graph indicate the standard deviation for each triplicate treatment. Statistical analyses were performed using t-tests (**P < 0.01).

To uncover the role of *LtuTPS32* in terpenoid biosynthesis, we cloned *LtuTPS32* and overexpressed it in tobacco for functional characterization. lRemarkably, the transgenic tobacco line OE2 exhibited flowering just 15 days post-cultivation. In contrast, the wild-type tobacco showed no flowering signs at this stage (**Supplementary Figure S3**). To unravel the underlying mechanisms and the potential role of LtuTPS32, we conducted RT-qPCR analyses focusing on the tobacco flowering gene *FT* and two key enzymes involved in the terpenoid biosynthesis pathway, geranylgeranyl diphosphate synthase (GGPPS) and geranyl diphosphate synthase (GPPS) (**Figure 9**). Expression levels of the *FT* gene in the transgenic lines were substantially elevated, with the OE2 line showing a 44.7-fold increase over the wild type. Similarly, expression in OE11 and OE13 lines was increased by 10.8-fold and 14.5-fold, respectively. These results suggest that the enhanced *FT* expression may underlie the early flowering phenotype. Additionally, we measured the expression of *GPPS* and *GGPPS*, finding increases of 2.8 to 3.4 times for GPPS and 7 to 13.2 times for GGPPS across the transgenic lines compared to the wild type. These enhanced levels imply that LtuTPS32 overexpression may boost the activity of these critical biosynthetic enzymes.

**Figure 10:**
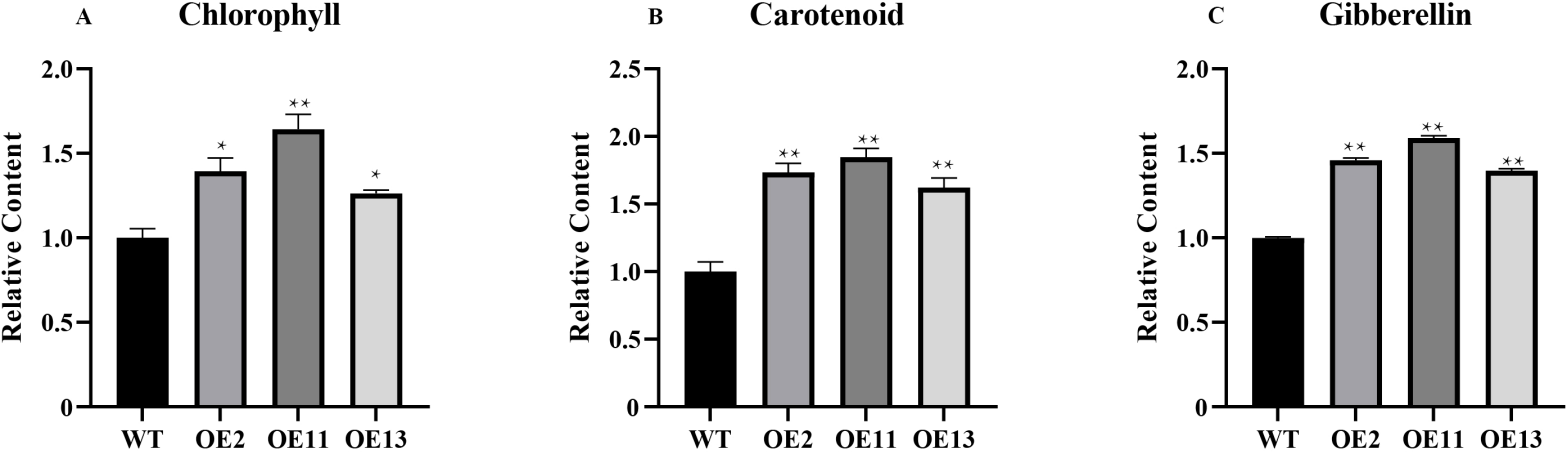
Determination of chlorophyll and carotenoid and gibberellin content in tobacco. **(A)** The relative content of chlorophyll in transgenic and wide-type plants. **(B)** The relative content of carotenoid in transgenic and wide-type plants. **(C)** The relative content of gibberellin in transgenic and wide-type plants. Error bars on the graph indicate the standard deviation for each triplicate treatment. Statistical analyses were performed using t-tests (*P < 0.05, **P < 0.01).

Chlorophyll, carotenoids, and gibberellins are common terpenoids, and they play a role in plant growth and development (Zhou et al., 2019; Camut et al., 2021; Wang et al., 2021). We also quantified the secondary metabolites impacted by terpenoid biosynthesis, namely chlorophyll, carotenoids, and gibberellins, in both transgenic and wild-type plants (**Figure 10**). The chlorophyll content was significantly higher in transgenic lines, with increases ranging from 58% to 104% over wild-type levels. Similarly, gibberellin content showed increments of 40% to 59%, correlating with the observed phenotypic changes.

## 4 Discussion

### 4.1 Expansion and evolution of the TPS family

The Terpene Synthase (TPS) gene family, since its initial discovery, has emerged as a cornerstone in plant molecular biology due to its remarkable diversity and structural complexity across various species. This family’s evolutionary journey is marked by significant variations, underpinning its crucial roles in an array of biological processes. A defining characteristic of *TPS* genes is their propensity for tandem repeats within plant genomes, a pattern that contrasts with segmental or whole-genome duplications and often reveals distinct lineage-specific evolutionary paths (Hanada et al., 2008; Freeling, 2009; Yang et al., 2022). For instance, in the grapevine (*Vitis vinifera*) genome, 45 tandemly duplicated *TPS* genes span a 680 kb region on chromosome 18, illustrating a remarkable genomic arrangement. Similarly, in rice (*Oryza sativa*), a cluster of 14 *TPS* genes extends over a 480 kb region on chromosome 4, further exemplifying the structural diversity within this gene family (Martin et al., 2010; Chen et al., 2011).

In this study, using the latest *L. chinense* genome annotation and *L. tulipifera* genome, we identified 12 additional *TPS* genes in *L. chinense* genome compared with previous studies. This enhanced understanding suggests substantial segments of tandemly duplicated *TPS* genes on chromosomes 1 and 10 of *L. chinense* and *L. tulipifera*, respectively. These patterns of gene density are indicative of evolutionary events that have uniquely shaped the TPS family within this genus. Previous research has highlighted the TPS family’s sensitivity to environmental stressors and a tendency toward pseudogenization, which facilitates rapid genomic adaptation to diverse ecological pressures (Thibaud-Nissen et al., 2009; Zou et al., 2009). In *L. tulipifera*, a higher frequency of *TPS* gene tandem duplications compared to *L. chinense* suggests a post-divergence evolutionary trajectory favoring enhanced adaptability and stress resilience.

From an evolutionary view, *TPS* genes are believed to have originated from a common ancestral bifunctional *CPS/KS* gene. The TPS-c subfamily, deemed the ancestral lineage, shares close ties with the TPS-e and TPS-f subfamilies, with the latter evolving from TPS-e to form the hybrid TPS-e/f subfamily. The TPS-h subfamily, represents a unique evolutionary branch, predominantly characterized by the presence of both DxDD and DDxxD domains, indicative of their bifunctional origins and specialized roles in conifer metabolism. The TPS-d subfamily, encompassing TPS-d-1 (gymnosperm monoterpene synthases), TPS-d-2 (sesquiterpene synthases), and TPS-d-3 (diterpene synthases), illustrates the functional diversity within this gene family, each subgroup distinguished by unique gene structures and lengths.

In angiosperms, the TPS-a, TPS-b, and TPS-g subfamilies, composed exclusively of genes involved in the biosynthesis of specific terpene compounds, play pivotal roles in plant-environment interactions. The TPS-a subfamily, primarily consisting of sesquiterpene synthases, is further divided into monocot-specific TPS-a-1 and eudicot-specific TPS-a-2. The TPS-b subfamily, mirroring the function of TPS-d-1 in gymnosperms, includes monoterpene or isoprene synthases. The TPS-g subfamily, initially characterized by its monoterpene synthases, has been shown in *Arabidopsis*, rice, and grape to also produce a spectrum of terpenes, including acyclic monoterpenes, sesquiterpenes, and diterpenes (Greenhagen et al., 2006; Tholl, 2006; O’Maille et al., 2008).

Our analysis of the *Liriodendron* TPS family aligns with these findings, predominantly identifying members of the TPS-a and TPS-b subfamilies, consistent with the eudicot classification of *Liriodendron*. This comprehensive analysis contributes significantly to our understanding of the *TPS* gene family’s evolutionary history and functional diversification in *Liriodendron*, shedding light on the intricate relationship between genetic evolution and ecological adaptation.

### 4.2 Structure diversity of *TPS* gene

Terpenoids, also known as isoprenoids, encompass a diverse array of compounds synthesized from the isomeric five-carbon building blocks, IPP and DMAPP. In plants, these compounds are produced through two distinct and independent biosynthetic pathways: the MEP pathway, localized in plastids, and the MVA pathway, functioning within the cytosol and peroxisomes. Both pathways converge in the synthesis of IPP and DMAPP, serving as the foundational substrates for the vast and varied terpenoid family. The remarkable diversity of terpenes found in plants can be attributed to several unique factors intrinsic to this class of chemicals. Firstly, the enzymes known as TPSs, which utilize prenyl diphosphates to form the basic terpene skeleton, often yield multiple products from a single substrate (Chen et al., 2011). Secondly, the terpene synthesis process is highly sensitive to even minor alterations in enzyme structure, a single amino acid change in a TPS can lead to significant variations in the terpene mix produced. Consequently, a solitary mutation at the pathway’s inception can spawn a multitude of new products. Additionally, the presence of *TPS* genes as a large gene family, typically comprising 30∼100 genes per genome, establishes an extensive framework for the evolution of novel terpenes through mutation and natural selection. Lastly, the basic terpene skeletons are further diversified through a range of modification reactions. These reactions are catalyzed by oxidative enzymes, methyltransferases, acyltransferases, prenyltransferases, and other enzymes characterized by their relaxed substrate and regiospecificity. This process effectively amplifies the total number of compounds produced, akin to combinatorial biochemistry, thereby contributing to the vast array of terpenoids observed in the plant kingdom (Pichersky and Raguso, 2018).

In our investigation, we successfully cloned the promoter of the *LtuTPS32* gene, revealing the presence of multiple cis-regulatory elements conducive to stress response: three abscisic acid-responsive elements (ABRE) and ten MYC elements. Subsequent *β*-glucuronidase (GUS) staining experiments demonstrated that the LtuTPS32 promoter could be robustly induced by abscisic acid (ABA) and methyl jasmonate (MeJA), suggesting a strong responsiveness to these phytohormones which are pivotal in plant stress signaling pathways. Notably, ABA is synthesized directly via the C15 pathway and indirectly via the C40 pathway in the chloroplast, cytosol, and mitochondria, and is considered a hormone closely associated with plant stress response (Leung and Giraudat, 1998; Nambara and Marion-Poll, 2003). MYC, a core transcription factor in the jasmonic acid (JA) signaling pathway, modulates plant reactions to JA and MeJA, which are crucial for plant growth, development, and environmental interaction (Creelman and Mullet, 1997; Blée, 1998; Wasternack and Hause, 2013). For instance, in apple (Malus domestica), JA is known to promote the germination of dormant buds and enhance the activity of alkaline lipase, facilitating the conversion of lipids into sugars, thus providing essential energy and nutrients for seedling growth. Throughout fruit development, increasing JA levels correlate with elevated ethylene synthesis, which promotes fruit ripening (Ranjan and Lewak, 1992). Moreover, the application of JA directly to leaves has been shown to suppress the expression of nuclear and chloroplast genes involved in photosynthesis, leading to decreased chlorophyll content and subsequent leaf yellowing, highlighting JA’s integral role in regulating plant senescence (Ueda et al., 1981; Weidhase et al., 1987). Additionally, experimental applications of MeJA have shown to increase the terpenoid content in the root callus of *Taraxacum officinale* and enhance the synthesis of triterpenoids in *Ganoderma lucidum*, indicating its significant role in secondary metabolite biosynthesis (Ren et al., 2010; Sharma and Zafar, 2016). These examples underscore the multifaceted roles of JA and MeJA in plant physiology, impacting everything from developmental processes and stress responses to the modulation of metabolic pathways critical for survival and adaptation.

### 4.3 Multiple functions of *TPS* genes

Terpenoids, the largest class of compounds in plants, are distributed across various tissues and play multifaceted roles. Mutations in genes such as *MCT*, *MDS*, *CMK*, *HDS*, and *HDR-1* within the terpenoid synthesis pathway in *Arabidopsis* have been linked to plant bleaching, downregulation of photosynthesis-related genes, and chloroplast developmental defects, highlighting their critical roles in plant physiology. Double mutants of *HMGR*1 and *HMGR2* in *Arabidopsis* exhibit male gametophyte lethality, with 50% of microspores undergoing atrophy and ambiguous endoplasmic reticulum membranes (Suzuki et al., 2009). Particularly, a mutation in *GGPPS1* leads to seedling bleaching and embryo lethality, emphasizing its indispensable role in chlorophyll biosynthesis (Tholl, 2015).

Terpenoids also play crucial ecological roles, such as attracting pollinators and deterring herbivores with their volatile compounds. For instance, several tropical orchids emit 1,8-cineole, attracting bees as pollinators (Zimmermann et al., 2006). Conversely, studies have shown that coniferous trees release volatile terpenoids in response to insect attacks (Miller et al., 2005). Recent research has further elucidated their role under plant stress conditions. For example, *Arabidopsis*, traditionally considered a “non-emitter,” has been found to emit low levels of constitutive terpenes from its leaves, phloem, and even roots. Overexpression of isoprene synthase in *Arabidopsis* leads to increased isoprene emission under high temperatures, resulting in lower leaf temperatures in transgenic plants compared to wild types, confirming isoprene’s role in thermal protection (Rosenkranz et al., 2007; Sasaki et al., 2007). In recent years, the study of terpenoids in plant immunity has gained prominence. For example, pinene, besides serving as an aromatic substance to attract insect pollinators, can also trigger plant immunity, enhancing resistance to fungi (Riedlmeier et al., 2017). Additionally, terpenoids like (*E*) - Neolidol and 1,8-cineole have been recognized for triggering plant immunity and possessing antimicrobial properties, further underscoring their ecological importance (Lee et al., 2004; Chen S. et al., 2020).

Terpenoids serve as precursors to numerous plant hormones and structural substances, playing vital roles across various biological processes. Specifically, geranylgeranyl diphosphate (GGPP) is a well-studied precursor of gibberellins (GAs), essential for growth and development (Yamaguchi, 2008). The transformation of GGPP to *ent*-kaurene catalyzed by CPS and KS, and its subsequent conversion to GA by cytochrome P450 oxygenation. In rice, mutations in *TPS* genes within the terpenoid synthesis pathway leads to reduced GA levels and plant dwarfism (Sakamoto et al., 2004). Additionally, GGPP is crucial for chlorophyll biosynthesis in plastids, where it is converted to chlorophyll by geranylgeranyl reductase (GGR). Terpenoids also contribute to the structural integrity of cell membranes. Sterols, synthesized from oxidosqualene within the endoplasmic reticulum, are key components of cell membranes in fungi, animals, and plants, crucial for regulating membrane fluidity and permeability and facilitating interactions with membrane lipids and proteins[1] (Feng et al., 2023).

The cellular environment significantly influences gene functions, even within the same gene family. For example, in tomatoes, functional characterization and subcellular localization studies of the TPS family revealed distribution in plastids, cytoplasm, chloroplasts, and mitochondria. Similar variability in subcellular localization has been reported for GGPPS proteins in peppers and *Arabidopsis*. Additionally, distinct protein isoforms within a gene family, such as the long and short isoforms of FPPS1 in *Arabidopsis*, may localize differently—mitochondria and cytoplasm, respectively, impacting their functional roles (Cunillera et al., 1997; Beck et al., 2013; Wang et al., 2018). In this study, our subcellular localization analysis of LtuTPS32 in tobacco leaves showed a pronounced chloroplast localization, suggesting its involvement in chlorophyll biosynthesis. This insight underscores the complexity of terpenoid biosynthesis and its critical functions across different cellular compartments, highlighting the intricate link between subcellular localization and metabolic roles in plants.

Further, we developed an overexpression vector for the *LtuTPS32* gene and performed heterologous transformation in tobacco. The transgenic plants displayed early flowering, potentially due to the significantly elevated expression of the flowering time gene (*FT*) compared to wild-type plants. This suggests that overexpression of *LtuTPS32* might accelerate flowering. Moreover, the key genes *GPPS* and *GGPPS* showed significantly increased expression in the transgenic lines, leading to higher synthesis rates of chlorophyll, carotenoids, and gibberellins. And the increased gibberellins may partly attribute to the early flowering. Given that GGPP serves as a precursor for these compounds, the enhanced expression of the *LtuTPS32* gene likely elevated *GGPPS* activity, boosting the biosynthesis capabilities of these vital terpenoids in the transgenic tobacco.

These findings underscore the pivotal role of *LtuTPS32* in enhancing the biosynthesis of key terpenoids in tobacco, likely contributing to the regulation of growth and developmental processes. The substantial increases in chlorophyll, carotenoids, and gibberellins not only validate the functional impact of *LtuTPS32* but also highlight its potential utility for enhancing metabolic pathways in plants. This research provides foundational insights for future investigations aimed at exploiting terpenoid biosynthesis pathways in *L. tulipifera* and other species for agricultural and biotechnological applications.

## Data Availability

The original contributions presented in the study are included in the article/**Supplementary Material**. Further inquiries can be directed to the corresponding authors.

## References

[B1] Aubourg S. Lecharny A. Bohlmann J. (2002). Genomic analysis of the terpenoid synthase (*AtTPS*) gene family of *Arabidopsis thaliana* . Mol. Genet. Genom. 267, 730–745. doi: 10.1007/s00438-002-0709-y 12207221

[B2] Bailey T. L. Johnson J. Grant C. E. Noble W. S. (2015). The MEME suite. Nucleic Acids Res. 43, 39–49. doi: 10.1093/nar/gkv416 PMC4489269 25953851

[B3] Beck G. Coman D. Herren E. Ruiz-Sola M. A. Rodríguez-Concepción M. Gruissem W.. (2013). Characterization of the *GGPP* synthase gene family in *Arabidopsis thaliana* . Plant Mol. Biol. 82, 393–416. doi: 10.1007/s11103-013-0070-z 23729351

[B4] Blée E. (1998). Phytooxylipins and plant defense reactions. Prog. Lipid Res. 37, 33–72. doi: 10.1016/S0163-7827(98)00004-6 9764311

[B5] Campos N. Rodríguez-Concepción M. Sauret-Güeto S. Gallego F. Lois L. M. Boronat A. (2001). Escherichia coli engineered to synthesize isopentenyl diphosphate and dimethylallyl diphosphate from mevalonate: a novel system for the genetic analysis of the 2-C-methyl-d-erythritol 4-phosphate pathway for isoprenoid biosynthesis. Biochem. J. 353, 59–67. doi: 10.1042/bj3530059 11115399 PMC1221543

[B6] Camut L. Gallova B. Jilli L. Sirlin-Josserand M. Carrera E. Sakvarelidze-Achard L.. (2021). Nitrate signaling promotes plant growth by upregulating gibberellin biosynthesis and destabilization of DELLA proteins. Curr. Biol. 31, 4971–4982.e4. doi: 10.1016/j.cub.2021.09.024 34614391

[B7] Cao Z. J. Ma Q. X. Weng Y. H. Shi J. S. Chen J. H. Hao Z. D. (2023). Genome-wide identification and expression analysis of *TPS* gene family in *liriodendron chinense* . Genes 14, 770. doi: 10.3390/genes14030770 36981040 PMC10048281

[B8] Chaw S. M. Liu Y. C. Wu Y. W. Wang H. Y. Lin C. Y. I. Wu C. S.. (2019). Stout camphor tree genome fills gaps in understanding of flowering plant genome evolution. Nat. Plants. 5, 63–73. doi: 10.1038/s41477-018-0337-0 30626928 PMC6784883

[B9] Chen C. Chen H. Zhang Y. Thomas H. R. Frank M. H. He Y.. (2020). TBtools: an integrative toolkit developed for interactive analyses of big biological data. Mol. Plant 13, 1194–1202. doi: 10.1016/j.molp.2020.06.009 32585190

[B10] Chen F. Tholl D. Bohlmann J. Pichersky E. (2011). The family of terpene synthases in plants: a mid-size family of genes for specialized metabolism that is highly diversified throughout the kingdom. Plant J. 66, 212–229. doi: 10.1111/j.1365-313X.2011.04520.x 21443633

[B11] Chen S. Zhang L. Cai X. Li X. Xin Z. (2020). (E)-Nerolidol is a volatile signal that induces defenses against insects and pathogens in tea plants. Hort. Res. 7, 52. doi: 10.1038/s41438-020-0275-7 PMC7109047 32257238

[B12] Christianson D. W. (2017). Structural and chemical biology of terpenoid cyclases. Chem. Revl. 117, 11570–11648. doi: 10.1021/cr050286w PMC5599884 28841019

[B13] Creelman R. A. Mullet J. E. (1997). Biosynthesis and action of jasmonates in plants. Annu. Rev. Plant Physiol. Plant Mol. Biol. 48, 355–381. doi: 10.1146/annurev.arplant.48.1.355 15012267

[B14] Cunillera N. Boronat A. Ferrer A. (1997). The *Arabidopsis thaliana FPS1* gene generates a novel mRNA that encodes a mitochondrial farnesyl-diphosphate synthase isoform. J. Biol. Chem. 272, 15381–15388. doi: 10.1074/jbc.272.24.15381 9182568

[B15] Degenhardt J. Köllner T. G. Gershenzon J. (2009). Monoterpene and sesquiterpene synthases and the origin of terpene skeletal diversity in plants. Phytochemistry 70, 1621–1637. doi: 10.1016/j.phytochem.2009.07.030 19793600

[B16] Dong S. Liu M. Liu Y. Chen F. Yang T. Chen L.. (2021). The genome of Magnolia biondii Pamp. provides insights into the evolution of *Magnoliales* and biosynthesis of terpenoids. Hortic. Res. 8, 38. doi: 10.1038/s41438-021-00471-9 33642574 PMC7917104

[B17] El-Gebali S. Mistry J. Bateman A. Eddy S. R. Luciani A. Potter S. C.. (2019). The Pfam protein families database in 2019. Nucleic Acids Res. 47, 427–432. doi: 10.1093/nar/gky995 PMC6324024 30357350

[B18] Enfissi E. M. A. Fraser P. D. Lois L. M. Boronat A. Schuch W. Bramley P. M. (2005). Metabolic engineering of the mevalonate and non-mevalonate isopentenyl diphosphate-forming pathways for the production of health-promoting isoprenoids in tomato. Plant Biotechnol. J. 3, 17–27. doi: 10.1111/j.1467-7652.2004.00091.x 17168896

[B19] Estévez J. M. Cantero A. Reindl A. Reichler S. León P. (2001). 1-Deoxy-D-xylulose -5- phosphate synthase, a limiting enzyme for plastidic isoprenoid biosynthesis in plants. J. Biol. Chem. 276, 22901–22909. doi: 10.1074/jbc.M100854200 11264287

[B20] Feng W. Mehari T. G. Fang H. Ji M. Qu Z. Jia M.. (2023). Genome-wide identification of the geranylgeranyl pyrophosphate synthase (*GGPS*) gene family involved in chlorophyll synthesis in cotton. BMC Genom. 24, 176. doi: 10.1186/s12864-023-09249-w PMC10077690 37020266

[B21] Freeling M. (2009). Bias in plant gene content following different sorts of duplication: tandem, whole-genome, segmental, or by transposition. Annu. Rev. Plant Biol. 60, 433–453. doi: 10.1146/annurev.arplant.043008.092122 19575588

[B22] Greenhagen B. T. O’Maille P. E. Noel J. P. Chappell J. (2006). Identifying and manipulating structural determinates linking catalytic specificities in terpene synthases. Proc. Natl. Acad. Sci. U. S. A. 103, 9826–9831. doi: 10.1073/pnas.0601605103 16785438 PMC1502538

[B23] Hanada K. Zou C. Lehti-Shiu M. D. Shinozaki K. Shiu S. H. (2008). Importance of lineage-specific expansion of plant tandem duplicates in the adaptive response to environmental stimuli. Plant Physiol. 148, 993–1003. doi: 10.1104/pp.108.122457 18715958 PMC2556807

[B24] He S. A. Hao R. M. (1999). Study on the natural population dynamics and the endangering habitat of *Liriodendron chinense* in China. Acta Phytoecol. Sin. 23, 87–95. doi: 10.3321/j.issn:1005-264X.1999.01.012

[B25] Hoeven R. S. V. D. Monforte A. J. Breeden D. Tanksley S. D. Steffens J. C. (2000). Genetic control and evolution of sesquiterpene biosynthesis in Lycopersicon esculentum and L. hirsutum. Plant Cell. 12, 2283–2294. doi: 10.1105/tpc.12.11.2283 11090225 PMC150174

[B26] Hong G. J. Xue X. Y. Mao Y. B. Wang L. J. Chen X. Y. (2012). *Arabidopsis MYC2* interacts with DELLA proteins in regulating sesquiterpene synthase gene expression. Plant Cell. 24, 2635–2648. doi: 10.1105/tpc.112.098749 22669881 PMC3406894

[B27] Hu B. Jin J. Guo A. Y. Zhang H. Luo J. Gao G. (2015). GSDS 2.0: an upgraded gene feature visualization server. Bioinformatics 31, 1296–1297. doi: 10.1093/bioinformatics/btu817 25504850 PMC4393523

[B28] Huang M. Abel C. Sohrabi R. Petri J. Haupt I. Cosimano J.. (2010). Variation of herbivore-induced volatile terpenes among *arabidopsis* ecotypes depends on allelic differences and subcellular targeting of two terpene synthases, *TPS02* and *TPS031* . Plant Physiol. 153, 1293–1310. doi: 10.1104/pp.110.154864 20463089 PMC2899926

[B29] Hyodo H. Yamakawa S. Takeda Y. Tsuduki M. Yokota A. Nishitani K.. (2003). Active gene expression of a xyloglucan endotransglucosylase/hydrolase gene, *XTH9*, in inflorescence apices is related to cell elongation in *Arabidopsis thaliana* . Plant Mol. Biol. 52, 473–482. doi: 10.1023/A:1023904217641 12856951

[B30] Katoh K. Standley D. M. (2013). MAFFT multiple sequence alignment software version 7: improvements in performance and usability. Mol. Biol. Evol. 30, 772–780. doi: 10.1093/molbev/mst010 23329690 PMC3603318

[B31] Kliebenstein D. J. Kroymann J. Brown P. Figuth A. Pedersen D. Gershenzon J.. (2001). Genetic control of natural variation in *Arabidopsis* glucosinolate accumulation. Plant Physiol. 126, 811–825. doi: 10.1104/pp.126.2.811 11402209 PMC111171

[B32] Köllner T. G. Schnee C. Gershenzon J. Degenhardt J. (2004). The variability of sesquiterpenes emitted from two *Zea* mays cultivars is controlled by allelic variation of two terpene synthase genes encoding stereoselective multiple product enzymes. Plant Cell. 16, 1115–1131. doi: 10.1105/tpc.019877 15075399 PMC423204

[B33] Lee B. H. Annis P. C. Tumaalii F. Choi W. S. (2004). Fumigant toxicity of essential oils from the Myrtaceae family and 1,8-cineole against 3 major stored-grain insects. J. Stored. Prod. Res. 40, 553–564. doi: 10.1016/j.jspr.2003.09.001

[B34] Lescot M. Déhais P. Thijs G. Marchal K. Moreau Y. Van de Peer Y.. (2002). PlantCARE, a database of plant cis-acting regulatory elements and a portal to tools for in silico analysis of promoter sequences. Nucleic Acids Res. 30, 325–327. doi: 10.1093/nar/30.1.325 11752327 PMC99092

[B35] Letunic I. Bork P. (2021). Interactive Tree of Life (iTOL) v5: an online tool for phylogenetic tree display and annotation. Nucleic Acids Res. 49, 293–296. doi: 10.1093/nar/gkab301 PMC8265157 33885785

[B36] Letunić I. Khedkar S. Bork P. (2021). SMART: recent updates, new developments and status in 2020. Nucleic Acids Res. 49, 458–460. doi: 10.1093/nar/gkaa937 33104802 PMC7778883

[B37] Leung J. Giraudat J. (1998). Abscisic acid signal transduction. Annu. Rev. Plant Physiol. Plant Mol. Biol. 49, 199–222. doi: 10.1146/annurev.arplant.49.1.199 15012233

[B38] Li K. Q. (2013). Population genetic structure and molecular phylogeography of *Liriodendron* (Nanjing: Nanjing Forestry University).

[B39] Lichtenthaler H. K. (1999). The 1-deoxy-d xylulose-5-phosphate pathway of isoprenoid biosynthesis in plants. Annu. Rev. Plant Physiol. Plant Mol. Biol. 50, 47–65. doi: 10.1146/annur-ev.arplant.50.1.47 15012203

[B40] Livak K. J. Schmittgen T. D. (2001). Analysis of relative gene expression data using real-time quantitative PCR and the 2(-Delta Delta C(T)) Method. Methods 25, 402–408. doi: 10.1006/meth.2001.1262 11846609

[B41] Martin D. M. Aubourg S. Schouwey M. B. Daviet L. Schalk M. Toub O.. (2010). Functional annotation, genome organization and phylogeny of the grapevine (*Vitis vinifera*) terpene synthase gene family based on genome assembly, FLcDNA cloning, and enzyme assays. BMC Plant Biol. 10, 226. doi: 10.1186/1471-2229-10-226 20964856 PMC3017849

[B42] Miller B. Madilao L. L. Ralph S. Bohlmann J. (2005). Insect-induced conifer defense. White pine weevil and methyl jasmonate induce traumatic resinosis, *de novo* formed volatile emissions, and accumulation of terpenoid synthase and putative octadecanoid pathway transcripts in Sitka spruce. Plant Physiol. 137, 369–382. doi: 10.1104/pp.104.050187 15618433 PMC548866

[B43] Murashige T. Skoog F. (1962). A revised medium for rapid growth and bio assays with tobacco tissue cultures. Physiol. Plant 15, 473–497. doi: 10.1111/j.1399-3054.1962.tb08052.x

[B44] Nambara E. Marion-Poll A. (2003). ABA action and interactions in seeds. Trends Plant Sci. 8, 213–217. doi: 10.1016/S1360-1385(03)00060-8 12758038

[B45] O’Maille P. E. Malone A. Dellas N. Andes Hess B. Jr. Smentek L. Sheehan I.. (2008). Quantitative exploration of the catalytic landscape separating divergent plant sesquiterpene synthases. Nat. Chem. Biol. 4, 617–623. doi: 10.1038/nchembio.113 18776889 PMC2664519

[B46] Peñuelas J. Llusià J. (2002). Linking photorespiration, monoterpenes and thermotolerance in Quercus. New Phytol. 155, 227–237. doi: 10.1046/j.1469-8137.2002.00457.x

[B47] Pichersky E. Gang D. R. (2000). Genetics and biochemistry of secondary metabolites in plants: an evolutionary perspective. Trends Plant Sci. 5, 439–445. doi: 10.1016/s1360-1385(00)01741-6 11044721

[B48] Pichersky E. Raguso R. A. (2018). Why do plants produce so many terpenoid compounds? New Phytol. 220, 692–702. doi: 10.1111/nph.14178 27604856

[B49] Potter S. C. Luciani A. Eddy S. R. Park Y. López R. Finn R. D. (2018). HMMER web server: 2018 update. Nucleic Acids Res. 46, 200–204. doi: 10.1093/nar/gky448 PMC6030962 29905871

[B50] Price M. N. Dehal P. S. Arkin A. P. (2010). FastTree 2–approximately maximum-likelihood trees for large alignments. PloS One 5, e9490. doi: 10.1371/journal.pone.0009490 20224823 PMC2835736

[B51] Ranjan R. Lewak S. (1992). Jasmonic acid promotes germination and lipase activity in non-stratified apple embryos. Physiol. Plant 86, 335–339. doi: 10.1034/j.1399-3054.1992.860222.x

[B52] Ren A. Qin L. Shi L. Dong X. Mu D. S. Li Y. X.. (2010). Methyl jasmonate induces ganoderic acid biosynthesis in the basidiomycetous fungus Ganoderma lucidum. Bioresour. Technol. 101, 6785–6790. doi: 10.1016/j.biortech.2010.03.118 20395130

[B53] Riedlmeier M. Ghirardo A. Wenig M. Knappe C. Koch K. Georgii E.. (2017). Monoterpenes support systemic acquired resistance within and between plants. Plant Cell. 29, 1440–1459. doi: 10.1105/tpc.16.00898 28536145 PMC5502447

[B54] Rodríguez-Concepción M. (2004). The MEP pathway: a new target for the development of herbicides, antibiotics and antimalarial drugs. Curr. Pharm. Des. 10, 2391–2400. doi: 10.2174/1381612043384006 15279616

[B55] Rosenkranz M. Gilmer F. Fischbach R. J. Sörgel C. Bachl A. Walter A.. (2007). Arabidopsis, a model to study biological functions of isoprene emission? Plant Physiol. 144, 1066–1078. doi: 10.1104/pp.107.098509 17468218 PMC1914154

[B56] Sakamoto T. Miura K. Itoh H. Tatsumi T. Ueguchi-Tanaka M. Ishiyama K.. (2004). An overview of gibberellin metabolism enzyme genes and their related mutants in rice. Plant Physiol. 134, 1642–1653. doi: 10.1104/pp.103.033696 15075394 PMC419838

[B57] Sapir-Mir M. Mett A. Belausov E. Tal-Meshulam S. Frydman A. Gidoniet D.. (2008). Peroxisomal localization of *Arabidopsis* isopentenyl diphosphate isomerases suggests that part of the plant isoprenoid mevalonic acid pathway is compartmentalized to peroxisomes. Plant Physiol. 148, 1219–1228. doi: 10.1104/pp.108.127951 18988695 PMC2577245

[B58] Sasaki K. Saito T. Lämsä M. Oksman-Caldentey K. M. Suzuki M. Ohyama K.. (2007). Plants utilize isoprene emission as a thermotolerance mechanism. Plant Cell Physiol. 48, 1254–1262. doi: 10.1093/pcp/pcm104 17711876

[B59] Schuhr C. A. Radykewicz T. Sagner S. Latzel C. Zenk M. H. Arigoni D.. (2003). Quantitative assessment of crosstalk between the two isoprenoid biosynthesis pathways in plants by NMR spectroscopy. Phytochem. Rev. 2, 3–16. doi: 10.1023/B:PHYT.0000004180.25066.62

[B60] Sharma K. Zafar R. (2016). Optimization of methyl jasmonate and β-cyclodextrin for enhanced production of taraxerol and taraxasterol in (Taraxacum officinale Weber) cultures. Plant Physiol. Biochem. 103, 24–30. doi: 10.1016/j.plaphy.2016.02.029 26950922

[B61] Suzuki M. Nakagawa S. Kamide Y. Kobayashi K. Ohyama K. Hashinokuchi H.. (2009). Complete blockage of the mevalonate pathway results in male gametophyte lethality. J. Exp. Bot. 60, 2055–2064. doi: 10.1093/jxb/erp073 19363204 PMC2682496

[B62] Takahashi S. Koyama T. (2006). Structure and function of cis-prenyl chain elongating enzymes. Chem. Rec. 6, 194–205. doi: 10.1002/tcr.20083 16900467

[B63] Thibaud-Nissen F. Ouyang S. Buell C. R. (2009). Identification and characterization of pseudogenes in the rice gene complement. BMC Genom. 10, 317. doi: 10.1186/1471-2164-10-317 PMC2724416 19607679

[B64] Tholl D. (2006). Terpene synthases and the regulation, diversity and biological roles of terpene metabolism. Curr. Opin. Plant Biol. 9, 297–304. doi: 10.1016/j.pbi.2006.03.014 16600670

[B65] Tholl D. (2015). Biosynthesis and biological functions of terpenoids in plants. Adv. Biochem. Eng. Biotechnol. 148, 63–106. doi: 10.1007/10_2014_295 25583224

[B66] Tholl D. Chen F. Petri J. Gershenzon J. Pichersky E. (2005). Two sesquiterpene synthases are responsible for the complex mixture of sesquiterpenes emitted from *Arabidopsis* flowers. Plant J. 42, 757–771. doi: 10.1111/j.1365-313X.2005.02417.x 15918888

[B67] Tu Z. Hao Z. Zhong W. Li H. (2019). Identification of suitable reference genes for RT-qPCR assays in *liriodendron chinense* (Hemsl.) sarg. Forests 10, 441. doi: 10.3390/f10050441

[B68] Tu Z. Shen Y. Wen S. Liu H. Wei L. Li H. (2021). A Tissue-Specific Landscape of Alternative Polyadenylation, lncRNAs, TFs, and Gene Co-expression Networks in *Liriodendron chinense* . Front. Plant Sci. 12. doi: 10.3389/fpls.2021.705321 PMC8343429 34367224

[B69] Ueda J. Kato J. Yamane H. Takahashi N. (1981). Inhibitory effect of methyl jasmonate and its related compounds on kinetin-induced retardation of oat leaf senescence. Physiol. Plant 52, 305–309. doi: 10.1111/j.1399-3054.1981.tb08511.x

[B70] Wang J. Y. Lin P. Y. Al-Babili S. (2021). On the biosynthesis and evolution of apocarotenoid plant growth regulators. Semin. Cell Dev. Biol. 109, 3–11. doi: 10.1016/j.semcdb.2020.07.007 32732130

[B71] Wang Q. Huang X. Q. Cao T. J. Zhuang Z. Wang R. Lu S. (2018). Heteromeric Geranylgeranyl Diphosphate Synthase Contributes to Carotenoid Biosynthesis in Ripening Fruits of Red Pepper (*Capsicum annuum* var. *conoides*). J. Agric. Food Chem. 66, 11691–11700. doi: 10.1021/acs.jafc.8b04052 30339374

[B72] Wasternack C. Hause B. (2013). Jasmonates: biosynthesis, perception, signal transduction and action in plant stress response, growth and development. An update to the 2007 review in Annals of Botany. Ann. Bot. 111, 1021–1058. doi: 10.1093/aob/mct067 23558912 PMC3662512

[B73] Weidhase R. A. Kramell H. M. Lehmann J. Liebisch H. W. Lerbs W. (1987). Methyljasmonate-induced changes in the polypeptide pattern of senescing barley leaf segments. Plant Sci. 51, 177–186. doi: 10.1016/0168-9452(87)90191-9

[B74] Wilkins M. R. Gasteiger E. Bairoch A. Sanchez J. C. Williams K. L. Appel R. D.. (1999). Protein identification and analysis tools in the ExPASy server. Methods Mol. Biol. 112, 531–552. doi: 10.1385/1-59259-584-7:531 10027275

[B75] Wu H. N. Hao Z. Y. Tu Z. H. Zong. Y. X. Yang. L. C. (2023). Re-annotation of the *Liriodendron chinense* genome identifies novel genes and improves genome annotation quality. Tree Genet. Genomes. 19, 30. doi: 10.1007/s11295-023-01605-x

[B76] Xiao M. (2019). Insight into the Molecular Basis of Ocimene-primed Plant Defense (Nanjing: Hunan Agricultural University).

[B77] Yamaguchi S. (2008). Gibberellin metabolism and its regulation. Annu. Rev. Plant Biol. 59, 225–251. doi: 10.1146/annurev.arplant.59.032607.092804 18173378

[B78] Yang L. Liu H. Hao Z. Zong Y. Xia H. Shen Y.. (2021). Genome-wide identification and expression analysis of *R2R3-MYB* family genes associated with petal pigment synthesis in *liriodendron* . Int. J. Mol. Sci. 22, 11291. doi: 10.3390/ijms222011291 34681950 PMC8538729

[B79] Yang P. Zhao H. Y. Wei J. S. Zhao Y. Y. Lin X. J. Su J.. (2022). Chromosome-level genome assembly and functional characterization of terpene synthases provide insights into the volatile terpenoid biosynthesis of *Wurfbainia villosa* . Plant J. 112, 630–645. doi: 10.1111/tpj.15968 36071028

[B80] Zhang C. G. Liu H. H. Hu S. Zong Y. X. Xia H. Li H. G. (2022). Transcriptomic profiling of the floral fragrance biosynthesis pathway of *Liriodendron* and functional characterization of the LtuDXR gene. Plant Sci. 314, 111124. doi: 10.1016/j.plantsci.2021.111124 34895551

[B81] Zhou S. Cheng X. Li F. Feng P. Hu G. Chen G.. (2019). Overexpression of *slOFP20* in tomato affects plant growth, chlorophyll accumulation, and leaf senescence. Front. Plant Sci. 10. doi: 10.3389/fpls.2019.01510 PMC6896838 31850017

[B82] Zhu S. H. (2020). The interspecific differences of the volatile components of floral scents in *Liriodendron* and its impacts on pollinators (Nanjing: Nanjing Forestry University).

[B83] Zimmermann Y. Roubik D. W. Eltz T. (2006). Species-specific attraction to pheromonal analogues in orchid bees. Behav. Ecol. Sociobiol. 60, 833–843. doi: 10.1007/s10886-010-9821-3

[B84] Zou C. Lehti-Shiu M. D. Thibaud-Nissen F. Prakash T. Buell C. R. Shiu S. H. (2009). Evolutionary and expression signatures of pseudogenes in *Arabidopsis* and rice. Plant Physiol. 151, 3–15. doi: 10.1104/pp.109.140632 19641029 PMC2736005

