# Supplementary figures and images for: Overexpression of the Liriodendron tulipifera TPS32 gene in tobacco enhances terpenoid compounds synthesis
Source: Front Plant Sci. 2024 Sep 17;15:1445103. doi: 10.3389/fpls.2024.1445103 (PMC11442295; doi:10.3389/fpls.2024.1445103)

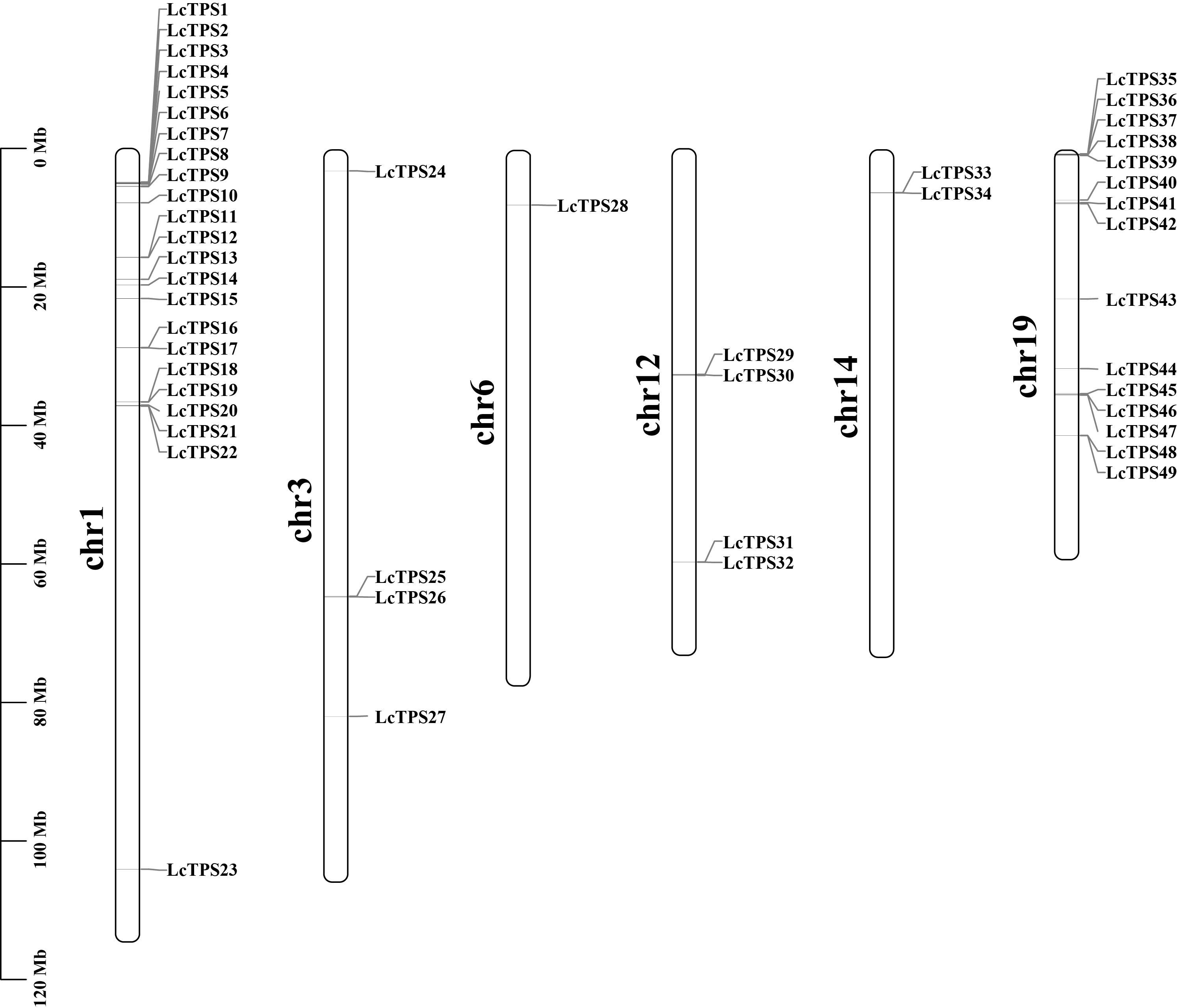

Supplement: Supplementary file 1 [file Image1.png]

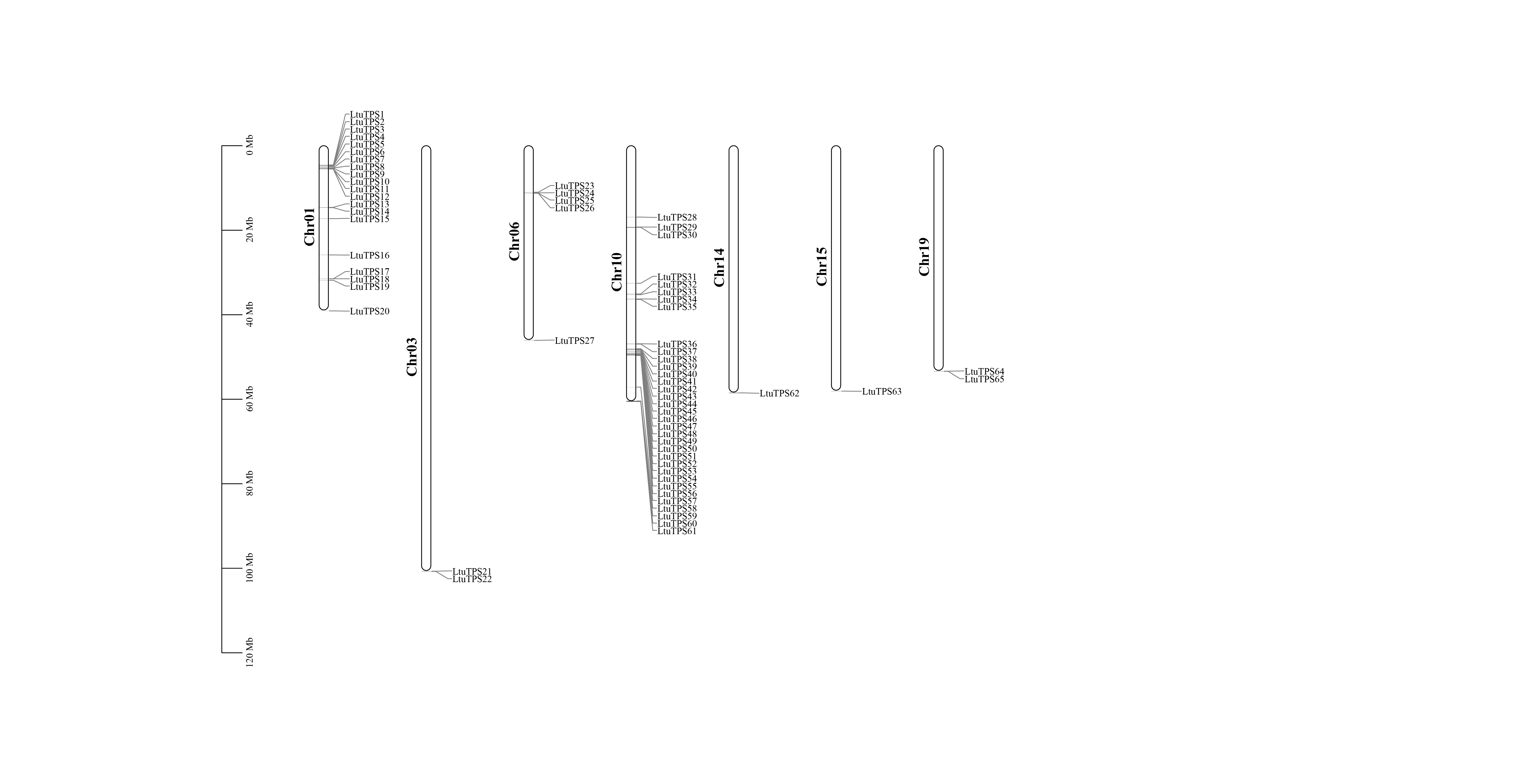

Supplement: Supplementary file 2 [file Image2.png]

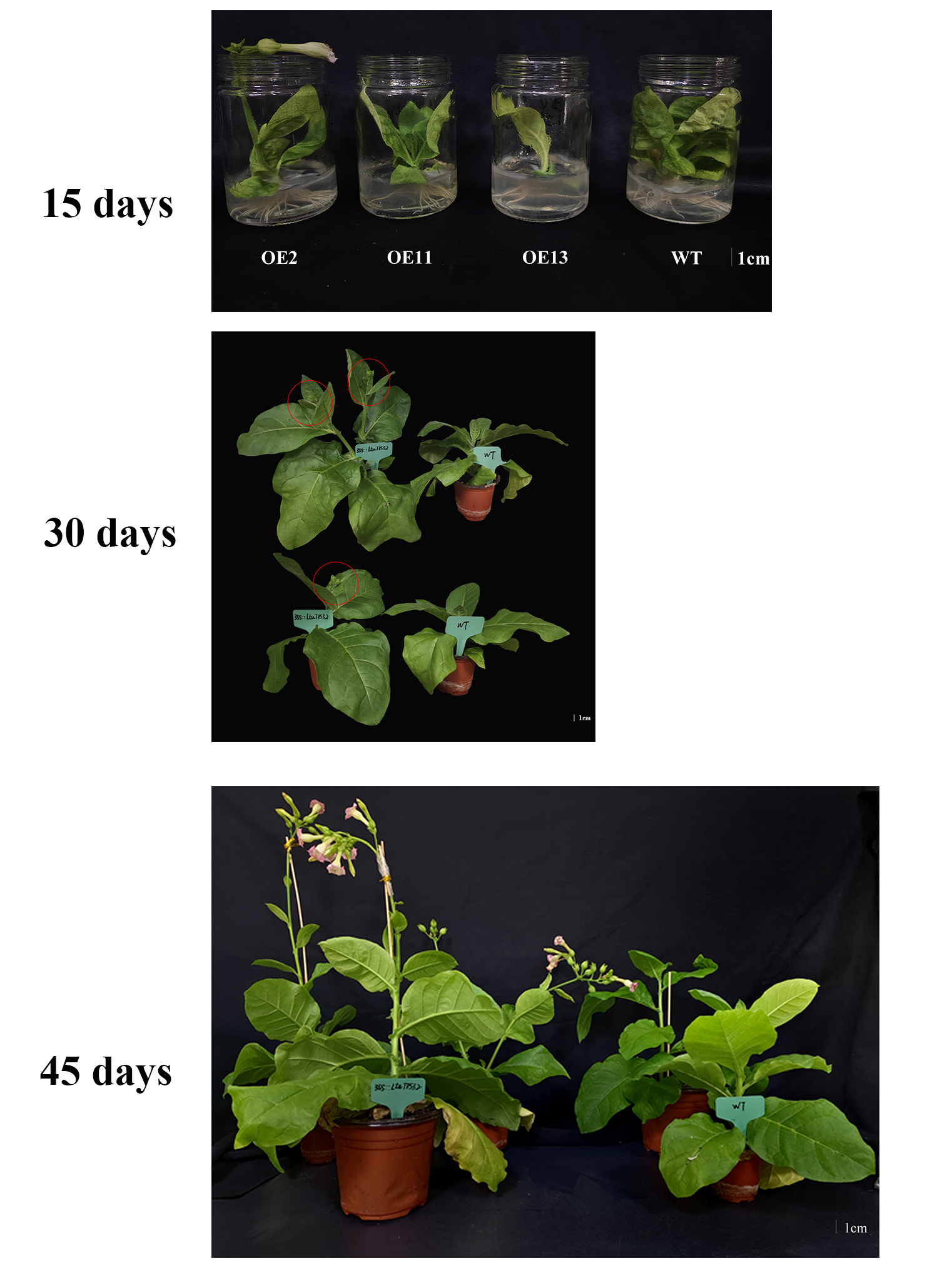

Supplement: Supplementary file 3 [file Image3.png]
